# Circadian rhythms of gut microbiota modulate nocturnal hyperglycemia in type 2 diabetes: a comprehensive review on mechanisms and chrono-targeted interventions

**DOI:** 10.3389/fmicb.2026.1834999

**Published:** 2026-06-17

**Authors:** Hanchi Ying, Qi Wang, Qian He, Yibo Cao, Guangyao Wei, Zhiqiang Xiao, Xiaoting Luo

**Affiliations:** 1 The First School of Clinical Medicine, Gannan Medical University, Ganzhou, China; 2 Department of Health Statistics, School of Public Health and Health Management, Gannan Medical University, Ganzhou, China; 3 College of Basic Medical Sciences, Gannan Medical University, Ganzhou, China

**Keywords:** biological clock, circadian rhythm, gut microbiota, nocturnal hyperglycemia, type 2 diabetes mellitus

## Abstract

Nocturnal hyperglycemia is a critical yet poorly controlled risk factor for complications in type 2 diabetes mellitus (T2DM), highlighting an unmet clinical need. The gut microbiota, a key regulator of host metabolism and circadian rhythms, has emerged as a pivotal player in this process, offering a novel therapeutic target. This article elaborates on the relationship between gut microbiota and nocturnal blood glucose control in T2DM from four key dimensions: the core gut microbiota-circadian clock, regulation by gut microbiota metabolites, the intestinal biological barrier, and neuroendocrine pathway mechanisms. Building on this, the article further explores gut microbiota-based intervention strategies: probiotic/prebiotic supplementation, time-restricted eating (TRE), and photobiomodulation therapy (PBMT). By integrating insights into the gut microbiota and circadian rhythms, this review proposes a novel therapeutic approach for patients with T2DM, offering concrete clinical guidance to improve nocturnal glycemic control.

## 1 Introduction

Type 2 diabetes mellitus (T2DM) is a metabolic disorder characterized by impaired glucose homeostasis. Beyond the conventional focus on average blood glucose levels, glycemic variability, particularly during the night, has garnered increasing attention with the widespread application of continuous glucose monitoring. For instance, over half of diabetic patients exhibit nocturnal hyperglycemia, which is significantly associated with all-cause mortality risk in this population (Cai et al., 2024). Current explanations for nocturnal glycemic dysregulation primarily focus on increased endogenous glucose production (EGP) during the night (Tuomi et al., 2016). However, existing hypoglycemic regimens show limited efficacy in controlling nocturnal hyperglycemia, suggesting their core mechanisms remain incompletely understood.

As the body’s “second genome,” the gut microbiota displays significant circadian oscillations in both its structure and function. These oscillations are coordinately regulated by multiple factors, including the host’s biological clock, feeding-fasting cycles, and environmental light exposure (Li J. et al., 2025). Microbiota rhythms not only influence the temporal synthesis of metabolites (e.g., short-chain fatty acids (SCFAs) and bile acids (BAs)) but also remotely modulate glucose metabolism through pathways such as the gut-liver and gut-brain axes. Patients with T2DM commonly exhibit disrupted microbial composition and circadian rhythm dysfunction, with impaired coupling between these two systems. These alterations suggest that nocturnal glucose abnormalities may reflect an overall imbalance in the “gut microbiota-host rhythm axis” (Thaiss et al., 2014). This article focuses on the association between gut microbiota and nocturnal hyperglycemia in T2DM. It examines the impact of circadian rhythms on the dynamics of the gut microbiota and related metabolic processes, to provide a theoretical foundation for developing prevention and management strategies for nocturnal hyperglycemia in T2DM.

## 2 Changes in composition and rhythm of gut microbiota in T2DM

Changes in the composition of the gut microbiota are closely linked to the development of T2DM. Multivariate metagenomic studies indicate significant differences in the overall diversity and composition of the gut microbiota between patients with T2DM and healthy individuals (Table 1). At the genus level, patients with T2DM consistently exhibit reduced abundance of SCFA-producing bacteria, including *Faecalibacterium prausnitzii* and *Bifidobacterium* (Snelson et al., 2021). Conversely, opportunistic pathogens, including *Escherichia coli*, specific *Prevotella* species, and certain *Lactobacillus strains*, are positively correlated with the risk of T2DM (Crudele et al., 2023). These alterations are accompanied by elevated systemic inflammatory markers, which further promote the proliferation of pathogenic bacteria. Furthermore, studies have revealed significant differences in gut microbiota composition between those with well-controlled and poorly controlled blood glucose (Wang et al., 2022). Specifically, among patients with T2DM, those with poor glycemic control show significantly increased abundance of gut bacteria, including members of the family *Enterobacteriaceae* and the genus *Enterococcus*. In contrast, the relative abundances of *Bifidobacterium*, *Bacteroides*, and *Lactobacillus* decrease significantly (XU et al., 2022). This body of evidence strongly suggests that specific alterations in the gut microbiota may be a key factor influencing nocturnal glycemic control and may represent an independent risk factor for nocturnal glucose dysregulation.

**Table 1:** Altered gut microbiota in T2DM across various studies.

References	Study subjects	Sequencing method	Abundance compared to control subjects
He et al. (2023)	29 T2DM patients and 16 healthy individuals	16S rRNA	*Lactobacillus*↑ *Clostridium*↓
Mei et al. (2024)	1851 T2DM patients, 2,770 prediabetes patients, 2,277 normoglycemic patients	Shotgun Metagenomes	*Clostridium bolteae*↑ *Clostridium citroniae*↑ *Escherichia coli*↑ *Bacteroides fragilis*↑ *Coprococcus eutactus*↓ *Faecalibacterium prausnitzii*↓ *Turicibacter sanguinis*↓ *Bacteroides plebeius*↓
Siptroth et al. (2023)	272 T2DM patients and 674 healthy individuals	16S rRNA	*Bacteroides*↑ *Blautia*↑ *Lachnoclostridium*↑ *Prevotella*↑ *Faecalibacterium*↓ *Lachnospira*↓ *Roseburia*↓ *Ruminococcus* **↓** *Coprococcus* ↓ *Subdoligranulum*↓
Wu and Park (2022)	551 T2DM patients and 3,378 healthy individuals	16S rRNA	*Bifidobacterium longum* ↑ *Prevotella copri* ↑ *Bacteroides uniformis* ↓ *Faecalibacterium* ↓ *Phocaeicola vulgatus* ↓ *Faecalibacterium prausnitzii* ↓
Kalkan et al. (2025)	24 T2DM patients and 10 healthy individuals	16S rRNA	*Lactobacillus* ↑ *Rothia* ↑ *Collinsella* ↑ *Eubacterium* ↑ *Bifidobacterium* ↑ *Blautia* ↑ *Faecalibacterium* ↓ *Faecalibacteriumprausnitzii* ↓
Carrizales-Sánchez et al. (2023)	21 children with T2DM and 20 healthy children	16S rDNA	*Faecalibacterium* ↑ *Prevotella* ↑ *Ruminococcus* ↓ *Bacteroides* ↓
Li X. et al. (2025)	508 T2DM patients and 1,538 healthy individuals	16S rRNA	*Victivallis* ↑ *Holdemanella* ↑ *Megasphaera* ↑ *Pseudofulvibacter* ↓ *Klebsiella* ↓ *Halomonas* ↓ *Butyrivibrio* ↓ *Prevotellamassilia* ↓
Sroka-Oleksiak et al. (2020)	22 T2DM patients and 27 healthy individuals	16S rRNA	*Escherichia* ↑ *Lactobacillus* ↑ *Bifidobacterium* ↓
Razavi et al. (2024)	18 T2DM patients and 18 healthy individuals	qPCR	*Bacteroidetes*↑ *Firmicutes*↓ *Actinobacteria*↓

From a temporal perspective, the abundance, composition, function, and metabolites of the gut microbiota are not static. Approximately 60% of the gut microbiota exhibits fluctuations in both amplitude and phase within 24 h (Thaiss et al., 2016; Thaiss et al., 2014), which are closely linked to the host’s feeding-fasting cycle, sleep–wake cycle, and intrinsic biological clock (Litichevskiy and Thaiss, 2022). A study on high-fat diet intervention in mice revealed that the circadian rhythmicity of SCFA-producing bacteria was lost, accompanied by the disappearance of circadian oscillations in SCFAs (Leone et al., 2015; Dantas Machado et al., 2022). Similarly, under T2DM conditions, the intensity of gut microbiota circadian oscillations diminishes, with multiple key bacterial genera exhibiting disrupted rhythms. Using a murine model of T2DM, Beli et al. (2019) demonstrated that diabetes leads to both reduced oscillation amplitude in genera such as *Akkermansia, Bifidobacterium*, and *Oscillospira*, and phase shifts in others, including *Prevotella* and *Proteobacteria*, potentially due to disrupted dietary patterns or dyssynchrony of the intestinal epithelial circadian clock (Reitmeier et al., 2020). Moreover, in mouse models with circadian disruption, gut microbiota dysrhythmia was found to further exacerbate glucose homeostasis imbalance and fat accumulation (Altaha et al., 2022). Furthermore, transplantation of the fecal microbiota from mice with lost circadian rhythmicity to germ-free mice resulted in the rapid development of glucose intolerance and obesity, supporting a causal contribution of disrupted microbial rhythms to metabolic disorders in experimental models (Thaiss et al., 2014). However, in human cohorts, current evidence mainly indicates associations between altered gut microbiota, arrhythmic microbiome signatures, glycemic traits, and T2DM risk, rather than definitive causality (Wang et al., 2022; Reitmeier et al., 2020).

While Table 1 summarizes the altered gut microbiota in T2DM across various studies, the reported microbial signatures are not completely consistent. These discrepancies may be partly explained by differences in study population characteristics, including age, ethnicity, geographical region, dietary pattern, obesity status, disease duration, glycemic control, and medication exposure. For example, antidiabetic drugs, particularly metformin, can independently reshape gut microbial composition and confound disease-associated microbial signatures (Forslund et al., 2015). Methodological heterogeneity may also contribute to divergent results, as the included studies used different sequencing or detection platforms, including 16S rRNA sequencing, shotgun metagenomics, and quantitative PCR, each with varying taxonomic resolution and analytical depth. In addition, variations in sample size, fecal sampling time, sample processing, bioinformatic pipelines, and statistical adjustment may further influence the identified taxa. For example, differences in the sampling site can alter local microenvironmental factors such as oxygen levels and pH. In the study by Sroka-Oleksiak et al. (2020), duodenal samples from the small intestine showed a microbiota composition dominated by *Lactobacillus*, which differed from the results obtained from large intestine samples. Therefore, the findings summarized in Table 1 should be interpreted as evidence of a broadly altered gut microbial ecosystem in T2DM rather than as a single uniform taxonomic pattern. Despite these inconsistencies, several studies converge on reduced abundance of SCFA-producing bacteria and relative enrichment of opportunistic or inflammation-associated taxa, supporting the biological relevance of gut microbial dysbiosis in T2DM.

## 3 Mechanisms underlying gut microbiota regulation of nocturnal blood glucose fluctuations

Metagenomic sequencing of fecal samples from T2DM patients revealed a close relationship between blood glucose fluctuations and diversity of the gut microbiota (Yan et al., 2022). These microbiota alterations are closely associated with disrupted microbial rhythms, altered metabolite production, impaired intestinal barrier function, low-grade inflammation, and abnormal neuroendocrine signaling, all of which may contribute to nocturnal glycemic dysregulation. In this context, the influence of the gut microbiota on nocturnal hyperglycemia should be understood as a multi-pathway process rather than the effect of a single bacterial taxon or metabolite. This interaction is also bidirectional. Long-term hyperglycemia may induce diabetic autonomic neuropathy, impair intestinal motility and rhythmicity, and thereby promote pathogenic bacterial overgrowth and further deterioration of gut microbiota structure (Kornum et al., 2025). This reciprocal relationship may partly explain why nocturnal glucose control in T2DM is difficult to improve through single-target interventions.

### 3.1 Gut microbiota-circadian rhythm-metabolism axis: the core hub of nocturnal glycemic dysregulation

#### 3.1.1 Bidirectional communication between host circadian rhythm and gut microbiota

The host’s circadian rhythm is maintained by the central biological clock in the hypothalamic suprachiasmatic nuclei (SCN) and by peripheral clocks in organs such as the intestine and liver. The core clock genes rely on brain and muscle arnt-like 1 (BMAL1) and circadian locomotor output cycles kaput (CLOCK) heterodimers to drive the cryptochromes (CRY1 and CRY2) and periods (PER1, PER2, PER3) in a negative feedback loop, thereby generating an autonomous oscillatory rhythm of approximately 24 h (Kavakli et al., 2022). Given the close regulation of the intestinal microenvironment by the host’s circadian clock, experimental studies have confirmed that Per2-knockout mice exhibit altered gut microbiota diversity and reduced total concentrations of SCFAs (Zhen et al., 2022). This is partly because the biological clock influences gut microbial metabolic activity by regulating feeding behavior (Tan et al., 2025; Sun et al., 2022). Strict dietary regulation establishes feeding-fasting cycles, providing periodic substrates for fermentation to the microbiota. This synchronizes metabolic activity with the host’s energy demands, ensuring circadian rhythmicity in the gut microbiome. Beyond feeding behavior, the host circadian clock indirectly influences the gut environment (e.g., oxygen levels, pH) by regulating intestinal motility, hormone secretion, and immune activity. For instance, circadian-regulated rhythmic secretion of BAs and mucus renewal in the intestinal epithelium influence microbial colonization and nutrient utilization. Furthermore, peripheral clocks influence gut microbiota abundance by modulating vagal nerve function, thereby shaping the microbial metabolic environment (Zielinski and Gibbons, 2022; Litichevskiy and Thaiss, 2022; Ding and Xiao, 2020).

Beyond passive regulation, the gut microbiota acts as an endogenous circadian regulator. The metabolites derived from microbial fermentation function as essential non-photic zeitgebers. Research indicates that the gut microbiota and their metabolites modulate the host’s epigenetic pathways via histone modifications to influence circadian rhythms (Sharma et al., 2025). Furthermore, these compounds cross the intestinal barrier and, in response to cues such as feeding cycles, entrain and induce phase shifts in the expression of circadian clock genes within peripheral organs such as the liver (Wang et al., 2023b; Choi et al., 2021). For instance, unconjugated BAs can significantly alter the expression of liver-related circadian genes in mice (Govindarajan et al., 2016). Germ-free mice regain rhythmic expression of peripheral clock genes and clock-controlling genes after microbiota transplantation, further demonstrating the potent synchronizing effect of microbiota on the host clock (Altaha et al., 2022). Simultaneously, the microbiota itself exhibits metabolism-related circadian oscillations that interact with food-induced oscillators to regulate intestinal motility, thereby ensuring synchronization with nutrient arrival in the gut (Zhang et al., 2023; Frazier et al., 2023).

#### 3.1.2 Gut microbiota-circadian rhythm dysregulation in T2DM

**Figure 1:**
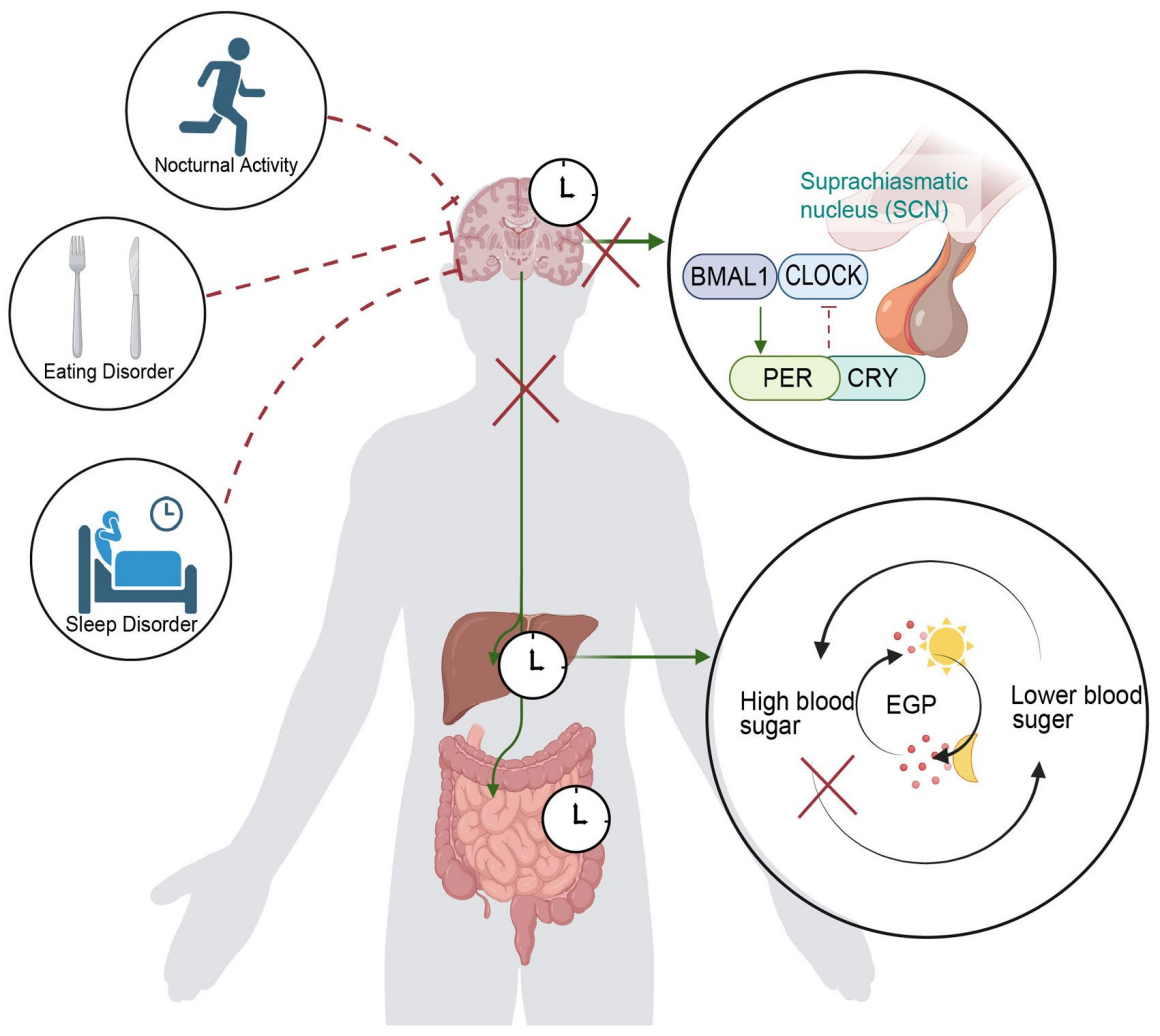
Disruption of the gut microbiota–circadian rhythm–metabolism axis under unhealthy lifestyle conditions. Under conditions such as sleep disturbances and irregular nocturnal activity, disruption of the core brain clock proteins (BMAL1/CLOCK) and the PER/CRY feedback loop impairs central signaling to peripheral biological clocks. This results in a shift in the nocturnal balance of hepatic gluconeogenesis toward hyperglycemia. BMAL1, Brain and muscle-like 1; CLOCK, Circadian locomotor output cycles kaput; PER, Period; CRY, Cryptochrome.

In healthy individuals, EGP exhibits a regular circadian rhythm: it increases during the nocturnal fasting period, while hepatic insulin sensitivity rises to suppress excessive gluconeogenesis, thereby maintaining stable fasting blood glucose levels. Around the time of awakening, insulin sensitivity and glucose uptake capacity peak, preparing the body for food intake (Peng et al., 2022). This metabolic rhythm is strictly dependent on the precise regulation of hepatic gluconeogenesis pathways and insulin signaling cascades by both central and peripheral circadian clocks, with the intestinal microenvironment providing critical peripheral signaling inputs to this regulatory process. However, studies have confirmed that, compared to healthy subjects, individuals with prediabetes exhibit a shift in the timing of blood glucose peaks toward the nighttime (Gubin et al., 2017). This nocturnal glycemic instability has also been confirmed in clinical settings. A study involving hospitalized patients with T2DM or new-onset hyperglycemia using a fully closed-loop insulin delivery system found that the day-to-day coefficient of variation of exogenous insulin requirements overnight was significantly higher than during any daytime period, indicating greater fluctuations in insulin sensitivity at night in T2DM patients (Boughton et al., 2021). This disturbance may be attributed to sleep disruption and irregular nocturnal activity, which are commonly observed in T2DM and desynchronize the endogenous rhythms of the gut microbiota from the host’s central circadian clock. First, disrupted circadian rhythms in patients with T2DM lead to abnormal BMAL1 expression. Decreased BMAL1 impairs β-cell function, thereby disrupting the expression of genes related to insulin secretion, endoplasmic reticulum function, and lipid metabolism, and altering the transcription of key hepatic gluconeogenic genes (Chen et al., 2023; Civelek et al., 2023). Furthermore, dysfunction of REV-ERBα/β genes in the central circadian clock under pathological conditions abolishes the physiological oscillations in insulin sensitivity and exacerbates nocturnal insulin resistance (Lucidi et al., 2023; Ding et al., 2021). More critically, dysrhythmia in the microbiota overlaps with host clock defects, creating a vicious cycle. Recent studies further reveal that gut microbiota-derived extracellular vesicles in T2DM accumulate abnormally at night and activate the hepatic gluconeogenic pathway, thereby directly disrupting the coordinated regulation between the circadian clock and hepatic glucose metabolism (Tan et al., 2025). Taken together, the core consequence of desynchronization within the gut microbiota-circadian rhythm-metabolism axis in T2DM is the failure of physiological inhibitory mechanisms governing nocturnal hepatic glucose output, which may represent an important contributor to the exacerbation of nocturnal hyperglycemia (Figure 1).

### 3.2 Gut microbiota metabolites

Gut microbiota metabolites, particularly SCFAs (acetate, propionate, and butyrate) and BAs, serve as key signaling molecules for communication within the gut microbiota-circadian rhythm-metabolism axis. Their production, secretion, and downstream signaling exhibit time-dependent patterns, although the reported peak phases differ across studies. In diurnal species, both fecal SCFA concentrations in pigs and serum butyrate levels in humans peak during the nocturnal rest period under fasting conditions, and these rhythms are stable. This suggests that the nighttime period represents a critical time window during which gut microbiota metabolism exerts key physiological effects (Wang et al., 2023b; Brignardello et al., 2022). In mice, SCFA peaks have been reported during the resting phase (daytime), whereas Tahara et al. (2018) observed peak SCFA concentrations during the active dark phase (Segers et al., 2019). These discrepancies may be related to species-specific activity patterns, sampling sites, diet composition, feeding schedules, and experimental models. Therefore, the rhythmic role of SCFAs should be interpreted less as a fixed peak time and more as a dynamic metabolic signal coupled to the feeding-fasting cycles and the circadian state of the host. More importantly, in the context of nocturnal hyperglycemia, the temporal pattern of SCFA signaling may be as biologically relevant as the absolute SCFA concentration. SCFAs can function as microbiota-derived timing cues that transmit information about nutrient availability and feeding-fasting cycles to peripheral metabolic clocks, thereby helping coordinate intestinal, pancreatic, and hepatic glucose-regulatory pathways across the 24-h cycle. Consistent with this interpretation, in chronic jetlag, the rhythm of SCFAs exhibits a phase delay, accompanied by weight gain, suggesting that altered SCFA timing may contribute to metabolic dysregulation (Desmet et al., 2021). Thus, disruption of SCFA rhythmicity may impair not only metabolite production but also the temporal alignment between microbial signals and host glucose homeostasis.

**Figure 2:**
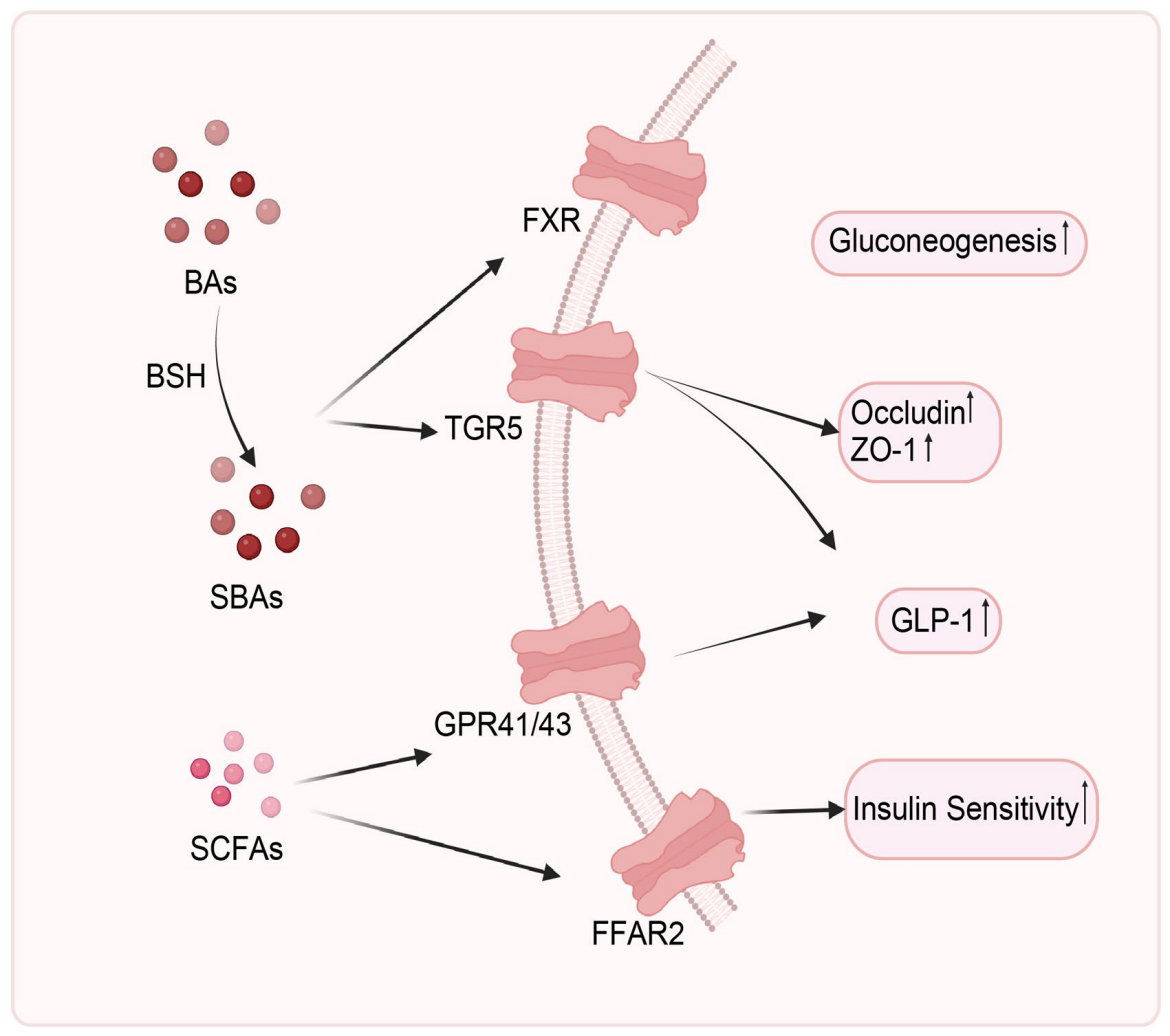
The role of gut dysbiosis-derived metabolites. SCFAs stimulate GLP-1 and insulin secretion by activating FFAR2 and GPR41/43. BAs are transformed into SBAs by bacterial BSH. SBAs then act on FXR and TGR5, synergizing with SCFAs to promote GLP-1 secretion, enhance intestinal barrier protein expression, and inhibit gluconeogenesis, thereby contributing to blood glucose stabilization. SCFAs, Short-chain fatty acids; GLP-1, Glucagon-like peptide-1; FFAR2, Free fatty acid receptor 2; GPR41/43, G-protein-coupled receptors 41/43; BAs, Bile acids; SBAs, Secondary bile acids; BSH, Bile salt hydrolase; FXR, Farnesoid x receptor; TGR5, Takeda G protein-coupled receptor 5.

The mechanism by which SCFAs stabilize blood glucose fluctuations partly stems from their activation of G-protein-coupled receptors (GPCRs) (e.g., GPR41, GPR43) on intestinal endocrine cells, which further promotes the secretion of glucagon-like peptide-1 (GLP-1). This regulates pancreatic β-cell function and insulin secretion via the cAMP/PKA pathway (Salehi et al., 2014; Secher et al., 2014). Furthermore, SCFAs serve as energy substrates. After uptake by the liver via the enterohepatic circulation, they regulate hepatic glucose and lipid metabolism. Additionally, SCFAs act directly on pancreatic β-cells through the free fatty acid receptor 2 (FFAR2) in peripheral tissues, contributing significantly to the maintenance of fasting blood glucose and overall glucose homeostasis (Rosli et al., 2023; Xie et al., 2025) (Figure 2).

From a circadian perspective, under physiological conditions, CRY, as a core component of the circadian clock, suppresses hepatic gluconeogenic gene expression. Under normal fasting conditions, CRY expression is temporally downregulated, allowing increased gluconeogenesis and blood glucose elevation to meet the body’s metabolic demands (Zhang et al., 2010). However, this increase is normally balanced by circadian regulation of hepatic insulin sensitivity and clock-controlled suppression of excessive gluconeogenesis (Peng et al., 2022). In T2DM, this temporal control is weakened, and nocturnal insulin sensitivity may fluctuate more markedly than during daytime periods (Boughton et al., 2021). Notably, in animal models, dietary interventions with fiber-rich foods (e.g., oat fiber) have been shown to restore circadian clock gene expression in the liver disrupted by high-fat feeding, an effect mediated by microbiota-derived SCFAs. *In vitro*, the addition of butyrate to hepatic cell models significantly affected the expression of BMAL1 and PER2 in a time-dependent manner (Leone et al., 2015; Tahara et al., 2018; Frazier and Chang, 2020). These findings indicate that SCFAs may participate in the entrainment of peripheral hepatic clocks rather than acting only as passive fermentation products. Because hepatic clock genes regulate key metabolic processes, including glycogen metabolism, gluconeogenesis, lipid handling, and insulin signaling, disruption of SCFA rhythms may lead to reduced or even complete loss of hepatic clock gene expression (Leone et al., 2015). In patients with T2DM, as discussed above, the reduced abundance of the SCFA-producing microbiota leads to significantly lower total SCFAs levels. Additionally, circadian disruption can induce phase shifts in SCFA rhythms. The former decreases the overall metabolic and anti-inflammatory benefits of SCFAs, whereas the latter disrupts the timing of SCFA-mediated signals required for nocturnal metabolic homeostasis. Together, these alterations may synergistically disrupt hepatic glucose output rhythms, resulting in relative hyperactivity of nocturnal hepatic glycogenolysis and gluconeogenesis, thereby exacerbating nocturnal hyperglycemia in patients with T2DM. Accordingly, the crosstalk between SCFAs and circadian rhythms helps illustrate how gut microbial dysrhythmia exacerbates glycemic dysregulation, particularly at night, in patients with T2DM.

Additionally, BA metabolism is dually regulated by both the host circadian rhythm and the gut microbiota. BAs serve as essential signaling molecules. In the intestine, BAs are deconjugated and transformed into secondary bile acids (SBAs) by gut bacteria expressing bile salt hydrolase (BSH). Approximately 95% of BAs are transported via the portal vein to hepatocytes, completing the enterohepatic circulation (Russell, 2003). BAs can regulate host glucose and lipid metabolism, among other processes, by activating the farnesoid X receptor (FXR) and the Takeda G protein-coupled receptor 5 (TGR5) (Gioiello et al., 2024). Activated TGR5 receptors enhance intestinal barrier integrity and stimulate GLP-1 secretion (Wang et al., 2023c; Shi et al., 2023). Additionally, BAs also exhibit a secretory rhythm whose circadian pattern closely matches feeding behavior. In humans, the circadian rhythm differences in BAs mainly originate from the asynchronous rhythms between conjugated and unconjugated BAs. Conjugated BAs peak after daytime meals due to gallbladder contraction following food intake, which facilitates bile release (Yang and Zhang, 2020). In contrast, unconjugated BAs produced in the gut reach their peak during deep sleep at night, a phenomenon that may enhance nighttime insulin sensitivity and help maintain nocturnal metabolic homeostasis (Al-Khaifi et al., 2018) (Figure 2). In induced T2DM mouse models, dysbiosis of BSH-expressing bacteria has been observed, leading to impaired BA conversion and disrupted circadian synchrony, consequently disturbing the temporal specificity of BA profiles and signaling pathways such as FXR/TGR5 (Bello et al., 2024; Tawulie et al., 2023). During sleep, when energy demands are low and circadian rhythms are more critical, this disruption in BA rhythms may more readily trigger reduced insulin sensitivity and inadequate suppression of hepatic gluconeogenesis. This promotes elevated nocturnal blood glucose levels and exacerbates diurnal hyperglycemia.

### 3.3 Gut barrier dysfunction and systemic inflammation

The intestinal barrier primarily comprises mechanical, chemical, immune, and biological components. The immune barrier consists mainly of immune cells within the intestinal mucosa, including various lymphocytes, while the gut microbiota forms the biological barrier. This barrier blocks harmful substances from entering the circulatory system by competitively excluding pathogenic bacterial colonization (Chen et al., 2021). Importantly, the intestinal barrier function is not a static defensive structure but a temporally organized system regulated by both host circadian clock and gut microbiota rhythms. In mouse models, the mRNA and protein levels of intestinal tight junction proteins (occludin and zonula occludens-1 (ZO-1)) oscillate in synchrony with the circadian cycle and correlate negatively with colonic permeability. Interestingly, intestinal barrier function in normal mice displays time-dependent changes over a 24-h period, with expression levels reaching their lowest point during the active phase to allow normal absorption of nutrients and other substances, whereas during the resting phase, barrier proteins are appropriately upregulated and permeability decreases, facilitating intestinal barrier recovery (Kyoko et al., 2014; Bishehsari et al., 2025). Genetic disruption of core clock genes—particularly BMAL1—abolishes this rhythmicity, leading to decreased expression of tight junction proteins, increased epithelial apoptosis, and enhanced intestinal permeability (Kyoko et al., 2014; Yang et al., 2023; Curtis et al., 2015). These findings indicate that intestinal barrier function is temporally organized rather than constant throughout the day, and that disruption of microbiota-clock synchrony may impair the time-dependent protection normally provided by the epithelial barrier.

Individuals with T2DM and insulin resistance exhibit metabolic endotoxemia, whereas healthy subjects do not. Under physiological conditions, lipopolysaccharides (LPS) derived from Gram-negative bacteria in the intestinal lumen cannot readily cross an intact intestinal mucosal barrier (Cani et al., 2007). Reduced production of SCFAs by beneficial bacteria, in synergy with circadian disruption, reduces the expression of intestinal tight junction proteins and weakens the rhythmic maintenance of epithelial barrier integrity. When this rhythmic barrier protection is impaired, intestinal permeability may increase, allowing LPS translocation, thereby converting local microbial dysbiosis into systemic inflammatory signaling. In this compromised state, LPS can enter the bloodstream through gaps in intestinal epithelial cells. By binding to receptors such as toll-like receptor 4 (TLR4), LPS further activates downstream inflammatory signaling pathways and induces the release of inflammatory mediators, including tumor necrosis factor-α (TNF-α) and interleukin-6 (IL-6). These mediators collectively trigger persistent systemic low-grade inflammation (Creely et al., 2007; Tilg and Moschen, 2008). From a temporal perspective, the inflammatory response to nighttime endotoxemia is significantly stronger than that during the daytime (Alamili et al., 2014). Therefore, the metabolic consequences of inflammation may be amplified during the nocturnal fasting period, when hepatic glucose production becomes a major determinant of blood glucose stability.

**Figure 3:**
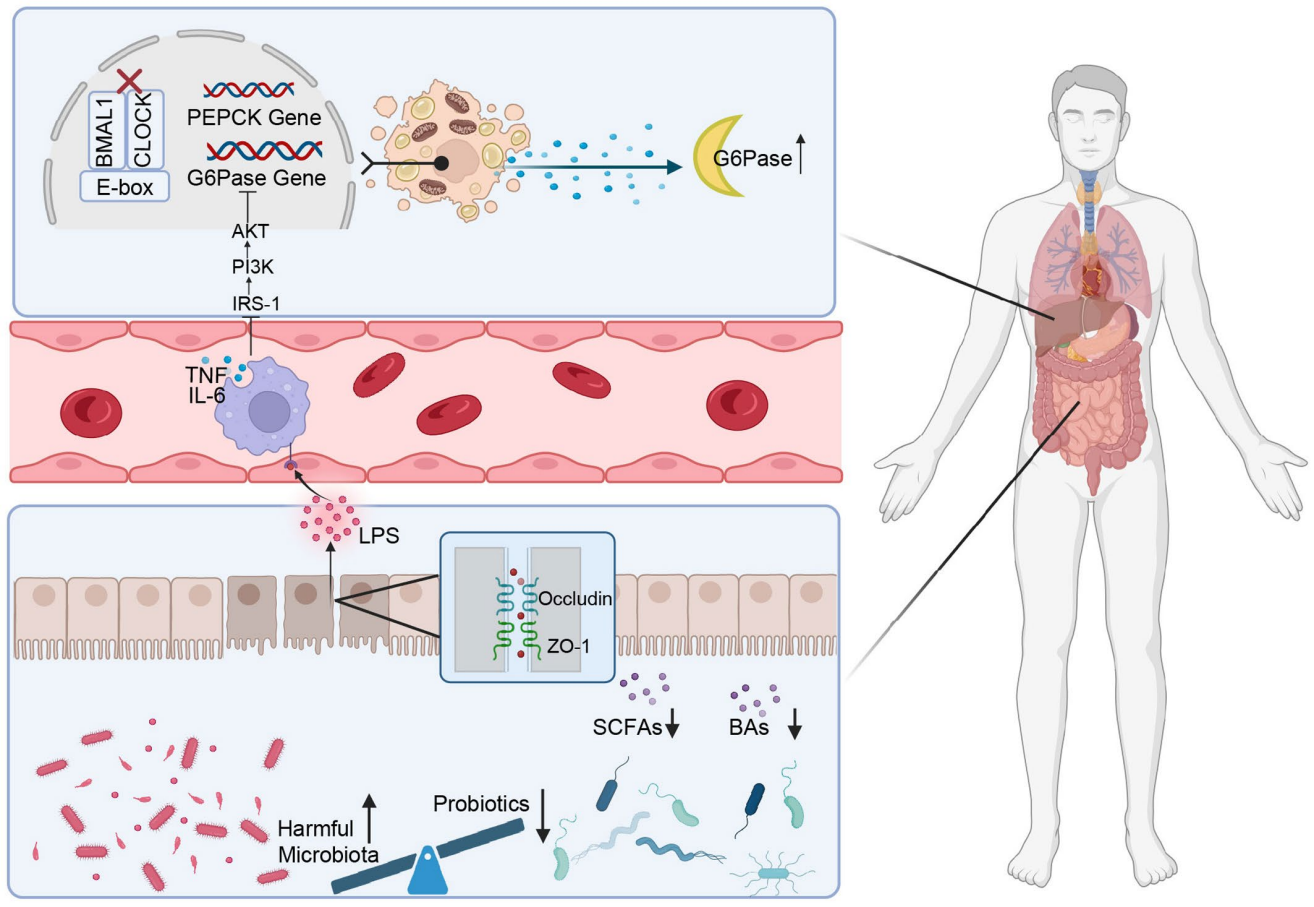
Disruption of the intestinal barrier and release of inflammatory factors drive nocturnal hyperglycemia. The pathological process involves three sequential steps: (1) Intestinal barrier dysfunction: Reduced production of microbiota-derived SCFAs and BAs downregulates intestinal tight junction proteins (occludin, ZO-1), leading to increased gut permeability. (2) Metabolic endotoxemia and inflammation: This facilitates the translocation of bacterial LPS into the circulation, triggering the hepatic and systemic release of pro-inflammatory cytokines such as TNF-α and IL-6. (3) Enhanced hepatic gluconeogenesis: These inflammatory signals subsequently upregulate key enzymes (e.g., PEPCK, G6Pase) in the hepatic gluconeogenic pathway, resulting in excessive glucose production, particularly during the nocturnal period. SCFAs, Short-chain fatty acids; BAs, Bile acids; ZO-1, Zonula occludens-1; LPS, Lipopolysaccharide; TNF-α, Tumor necrosis factor-alpha; IL-6, Interleukin-6; PEPCK, Phosphoenolpyruvate carboxykinase; G6Pase, Glucose-6-phosphatase.

Animal and cellular studies have demonstrated that inflammatory cytokines, such as TNF-α, impair insulin signaling. Specifically, TNF-α inhibits the tyrosine phosphorylation of the insulin receptor substrate-1, thereby disrupting the phosphatidylinositol 3-kinase (PI3K)/protein kinase B (AKT) pathway. This disruption attenuates the suppression of key hepatic gluconeogenic genes, such as glucose-6-phosphatase (G6Pase) and phosphoenolpyruvate carboxykinase (PEPCK), and interferes with normal insulin signal transduction, thus culminating in insulin resistance. During daytime feeding periods, postprandial insulin secretion and nutrient-driven metabolic responses may partially compensate for impaired insulin signaling. In contrast, during nocturnal fasting, blood glucose stability relies more heavily on the appropriate suppression of hepatic glycogenolysis and gluconeogenesis. Therefore, persistent LPS-induced inflammatory signaling may exert a more direct effect on nocturnal hyperglycemia by weakening insulin-mediated inhibition of hepatic glucose output. Against the backdrop of enhanced nocturnal hepatic gluconeogenesis, sustained release of inflammatory factors further suppresses glucose uptake and utilization in peripheral tissues. These combined effects ultimately lead to elevated nocturnal blood glucose levels (Hotamisligil, 2006; Meshkani and Adeli, 2009; Perry et al., 2015; Guo et al., 2025) (Figure 3). Thus, the temporal specificity of gut barrier dysfunction in T2DM lies not only in the circadian regulation of intestinal permeability itself, but also in the fact that inflammation-induced hepatic insulin resistance becomes metabolically critical during the nocturnal fasting window.

### 3.4 Neuroendocrine axis dysfunction

#### 3.4.1 The vagal nerve

The gut microbiota and the central nervous system form a tightly regulated network through the gut-brain axis. As the key communication pathway of the gut-brain axis, the vagal nerve exhibits pronounced circadian rhythmicity in its activity, reaching its peak physiological tone during nocturnal sleep periods due to parasympathetic dominance in humans. Regarding efferent pathways, the SCN regulates EGP via vagal nerve efferent fibers, thereby restraining excessive nocturnal blood glucose fluctuations and maintaining glucose stability in healthy individuals (Peng et al., 2022). On the afferent side, SCFAs produced by gut microbiota fermentation of fiber activate GPR41/43 on vagal nerve terminals (Borgmann and Fenselau, 2024), transmitting signals to brain regions such as the hypothalamus to regulate insulin sensitivity. However, impaired vagal function is a common finding in individuals with T2DM (Rinaldi et al., 2023). On the one hand, the vagal nerve lacks sufficient activation signals from gut metabolites, exacerbating nocturnal hyperglycemia (Zhang et al., 2025). Furthermore, chronic inflammation in T2DM impairs the vagal cholinergic anti-inflammatory pathway, which in turn further aggravates nocturnal insulin deficiency and insulin resistance (Han et al., 2025; Rahman et al., 2024).

#### 3.4.2 Melatonin (MT)

MT is traditionally recognized as a hormone secreted by the pineal gland. Its secretion displays a pronounced diurnal rhythm, with the primary release occurring at night in humans, typically peaking around 00:00 and 04:00 a.m.(Tordjman et al., 2017). As a key circadian rhythm-regulating hormone, it primarily modulates the host’s sleep–wake cycle. MT also participates in systemic glucose homeostasis regulation by activating melatonin receptors, which are highly expressed in pancreatic β-cells (Karamitri and Jockers, 2019; Sharma et al., 2015). Furthermore, MT promotes the tyrosine phosphorylation of insulin receptor and the insulin-like growth factor receptor, which maintains the survival and function of β-cells (Picinato et al., 2008).

Current research indicates that the gut is the primary site of extra-pineal MT secretion, with concentrations reaching up to 400 times those in the pineal gland (Iesanu et al., 2022). Unlike the endocrine nature of pineal MT, gut-derived MT primarily functions via paracrine mechanisms and maintains a close bidirectional regulatory relationship with the gut microbiota. On the one hand, MT in the gut can regulate bacterial genera and increase the abundance of relevant beneficial bacteria (Xu et al., 2025). Moreover, MT levels exhibit chronotype-dependent rhythmicity and can restore oscillations in the gut microbiota (Li W. et al., 2023). On the other hand, the metabolic state of the gut microbiota directly determines the efficiency of MT synthesis. As the precursor for MT synthesis, tryptophan is shunted by beneficial gut bacteria toward the serotonin (5-HT) and MT synthesis pathways. In the context of gut microbiota-circadian rhythm axis disruption, overgrowth of certain harmful bacteria in patients with T2DM may promote tryptophan loss via the kynurenine pathway, reducing total MT synthesis (Du et al., 2024), and induce phase shifts in MT secretion, manifested as secondary daytime peaks (Qian et al., 2022). A 12-year prospective study by McMullan et al. (2013) found that reduced nocturnal MT levels were independently associated with an increased risk of T2DM. MT functions as a potent anti-inflammatory molecule that preserves intestinal barrier integrity. Disruption of its normal nocturnal rhythm reduces MT synthesis, increasing systemic oxidative stress (Tuomi et al., 2016; Liu et al., 2024). Mechanistically, animal studies have demonstrated that MT deficiency markedly upregulates hepatic PEPCK expression, accelerates hepatic glucose production, and blunts insulin-mediated AKT phosphorylation (Nogueira et al., 2011). Collectively, reduced MT signaling exacerbates oxidative stress, impairs hepatic insulin sensitivity, and dysregulates gluconeogenesis—key drivers of nocturnal insulin resistance and hyperglycemia in T2DM.

There remains controversy regarding additional MT supplementation in metabolically impaired individuals. A systematic review and meta-analysis of 16 randomized controlled trials reported beneficial effects of MT supplementation on fasting blood glucose, insulin resistance, and glycated hemoglobin A1c (HbA1c), with most studies reporting no obvious adverse effects (Delpino et al., 2021). However, a randomized placebo-controlled crossover trial in men with T2DM showed that nightly supplementation with 10 mg of MT for three months was associated with reduced insulin sensitivity (Lauritzen et al., 2022). In addition, an *in vitro* study showed that MT impaired insulin sensitivity in human adipose tissue, with a more pronounced effect during the night (Zambrano et al., 2024). These apparently inconsistent findings may be explained by differences between physiological endogenous MT rhythms and pharmacological exogenous MT exposure. Reduced nocturnal endogenous MT may indicate circadian disruption and increased metabolic risk (Tuomi et al., 2016; McMullan et al., 2013), whereas exogenous MT supplementation may alter glucose regulation depending on dose, timing, treatment duration, and patient metabolic phenotypes (Rubio-Sastre et al., 2014). For example, high-dose nighttime MT may overlap with the physiological nocturnal decline in insulin sensitivity and further suppress insulin signaling in susceptible individuals (Lauritzen et al., 2022; Zambrano et al., 2024). In contrast, lower-dose or appropriately timed MT may be more likely to improve sleep and circadian alignment, thus indirectly benefiting glucose metabolism (Tordjman et al., 2017; Karamitri and Jockers, 2019). Furthermore, the timing of supplementation matters. Previous studies have shown that when taken at night under fasting conditions, MT can physiologically inhibit insulin secretion, allowing pancreatic β-cells to rest and recover, while also improving sleep and circadian synchronization, thereby exerting beneficial effects on glucose metabolism, including enhancing insulin sensitivity, protecting β-cell function, and improving long-term glycemic homeostasis. Conversely, when exogenous MT is taken during the daytime or after the evening meal, this inhibitory effect on insulin secretion impairs postprandial glucose clearance, increasing glycemic variability and the risk of T2DM (Garaulet et al., 2020). Therefore, MT should not be considered a uniformly beneficial metabolic intervention. Future clinical studies should stratify patients by baseline circadian status, sleep disturbance, glycemic phenotype, medication use, and endogenous MT rhythm, and compare different doses and administration times before MT can be recommended as an adjuvant strategy for nocturnal glycemic control.

#### 3.4.3 GLP-1

**Figure 4:**
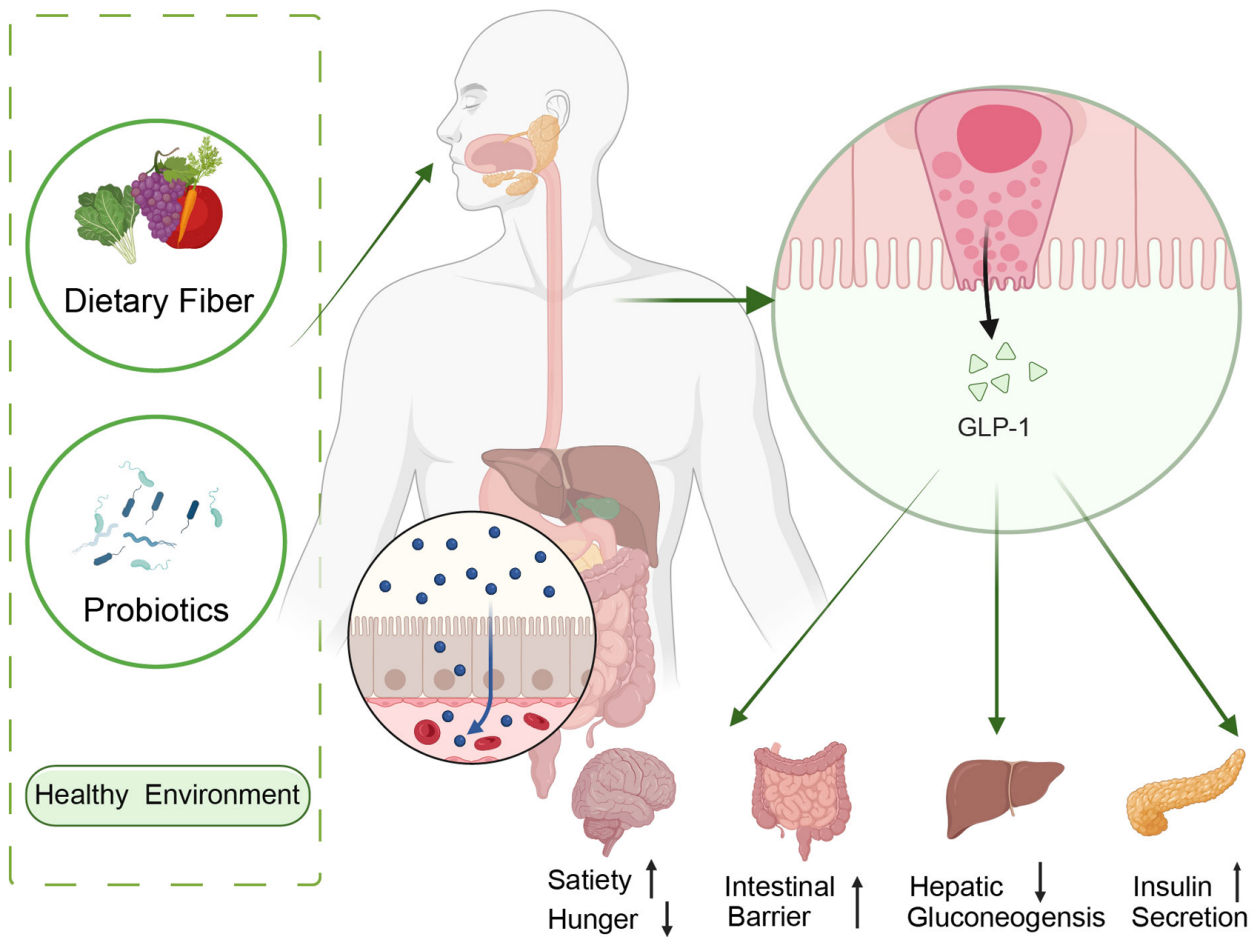
Multifunctional roles of GLP-1 in regulating glucose homeostasis. The main physiological effects of GLP-1 are appetite suppression, satiety enhancement, intestinal barrier improvement, inhibition of hepatic gluconeogenesis, and stimulation of insulin secretion. GLP-1, Glucagon-like peptide-1.

GLP-1 is secreted by both intestinal L cells and a discrete population of neurons in the nucleus tractus solitarius of the medulla oblongata (Kabahizi et al., 2022). As a key regulator of blood glucose homeostasis, GLP-1 exerts significant dual central and peripheral effects. Centrally, GLP-1 acts on the GLP-1 receptor (GLP-1R) neurons in the dorsomedial hypothalamus, predominantly GABAergic, directly reducing hunger and suppressing food intake (Kim et al., 2024; Park et al., 2025). Peripherally, GLP-1 enhances glucose-dependent insulin secretion from pancreatic β-cells (Stožer et al., 2021). The enteric vagal nerve serves as a bridge connecting peripheral GLP-1 to central regulation (Holst et al., 2022), and gut microbiota metabolites SCFAs directly stimulate L cells to secrete GLP-1, forming a cross-system mechanism. Furthermore, GLP-1 maintains intestinal barrier homeostasis by activating GLP-1R, upregulating the PI3K/AKT/HIF-1α pathway, promoting tight junction protein expression and reducing intestinal permeability. This decreases LPS transmucosal leakage and alleviates inflammatory insulin resistance (Wang et al., 2025) (Figure 4).

Notably, GLP-1 secretion itself exhibits significant circadian rhythmicity, and this rhythm is regulated by the gut microbiota. In germ-free mice, the GLP-1 secretory rhythm shifts toward the fasting period, whereas transplantation of fecal microbiota from normal mice restores the feeding-related secretory peak (Martchenko et al., 2020). However, a phenomenon distinct from that in mouse models occurs in patients with T2DM: the rhythmicity of GLP-1 secretion is markedly diminished, showing a reduced amplitude of circadian oscillations rather than a phase shift (Martchenko et al., 2021). Consequently, in T2DM, alterations in GLP-1 secretion primarily manifest as reduced satiety signals in central regions, leading to increased food cravings and disrupted energy intake rhythms during nocturnal fasting (Siemian et al., 2021). Additionally, such circadian disruption impairs the suppression of glucagon secretion, thereby exacerbating nocturnal gluconeogenesis (Tschöp et al., 2023). Meanwhile, impaired GLP-1-mediated intestinal barrier protection, coupled with dysregulated circadian rhythmicity of intestinal metabolites, further perturbs nocturnal metabolic homeostasis.

As discussed above, GLP-1 is not only a first-line glucose-lowering drug target for T2DM patients, but its potential role in restoring host circadian rhythms is also gaining increasing attention. In mouse models, intervention with GLP-1R agonist analogs alters hepatic clock gene expression (Ando et al., 2013). Therefore, based on chronobiological evidence, it is suggested that administration of exenatide (a GLP-1R agonist analog) in the morning can better restore the hepatic circadian clock, which is impaired by irregular eating patterns in humans (Xu et al., 2024). Although current studies focus primarily on animal models, they strongly suggest that, in clinical practice, the administration time of GLP-1R agonists may need to be individually adjusted according to the patient’s circadian rhythms. In conclusion, the imbalance in the intestinal microenvironment of T2DM compromises this critical nocturnal blood glucose “guardian” mechanism by impairing GLP-1 secretion and its circadian rhythm. As first-line glucose-lowering agents, GLP-1R agonists show promising potential in stabilizing nocturnal blood glucose fluctuations.

## 4 Intervention strategies targeting the “gut microbiota-circadian rhythm” axis

### 4.1 Probiotic/prebiotic supplementation

Probiotics are a class of live microorganisms beneficial to the host, whereas prebiotics are non-digestible compounds that are metabolized by gut microorganisms. Both exert physiological effects by regulating gut microbiota balance, enhancing immune function, promoting nutrient absorption, and improving outcomes in inflammatory diseases. Extensive research indicates that probiotic supplementation can restore gut microbiota balance, improve intestinal permeability, modulate immune function, and reduce pro-inflammatory cytokine production (Snelson et al., 2021; Toshimitsu and Irie, 2025; Li Y. et al., 2023). In addition, Cheng et al. (2020) demonstrated that specific prebiotics (e.g., beta-glucan and inulin) can reverse the phase advance of BMAL1 in the hypothalamus and suppress weight gain in mice with circadian disruption—without affecting dietary intake. This finding provides a stronger theoretical basis for the potential application of prebiotics in the management of T2DM and circadian rhythm disorders.

For patients with T2DM, multiple randomized controlled trials have reported improvements in fasting glucose, fasting insulin, and HbA1c following probiotic supplementation, with multi-strain probiotics showing significantly better glucose-lowering effects than single-strain probiotics (Zarezadeh et al., 2022; Zhang et al., 2022). However, for individual patients, nonspecific probiotic supplementation may have limited efficacy, as microbial effects depend largely on strain specificity, functional properties, and host physiological status. The Microbiome and Insulin Longitudinal Evaluation Study (MILES) showed that specific butyrate-producing bacteria, including taxa related to *Coprococcus* and *Oscillibacter*, were associated with more favorable insulin homeostasis, supporting the potential value of function-oriented microbial selection (Cui et al., 2022).

Beyond strain selection, the timing of administration may represent another critical determinant. The aforementioned animal studies (Cheng et al., 2020) support their circadian benefits, and an additional study in piglets demonstrated that dietary probiotics exert strictly circadian time-dependent regulation of the gut microbiota, increasing microbial α-diversity only at ZT16 (nighttime) (Luo et al., 2024). This suggests that the therapeutic efficacy of these interventions may be influenced by the timing of administration. For example, in a mouse model of chronic stress, inulin administered in the evening produced stronger anti-inflammatory effects than when administered in the morning (Alli et al., 2022). However, controlled clinical trials validating chrono-targeted probiotic or prebiotic dosing in patients with T2DM are currently lacking (Zhai et al., 2025). Therefore, timed probiotic or prebiotic administration should be regarded as a promising but unvalidated strategy. Future studies should investigate defined strains, dosing time, treatment durations, metabolic phenotypes, and nocturnal glycemic outcomes in controlled clinical settings.

### 4.2 Time-restricted eating

A growing body of animal and human studies confirms that the timing, frequency, and regularity of eating are closely linked to circadian rhythm disruption. It is known that circadian rhythm disruption can affect the expression of clock-regulating genes, the composition of the gut microbiota, and the production of microbial metabolites. The concept of time-restricted eating (TRE) originates from the regulatory role of feeding time on circadian rhythms. Specifically, TRE refers to a dietary approach in which food intake is restricted to a specific time window during the day without altering total daily caloric intake (Xie et al., 2024). In circadian rhythm-disrupted mouse models, TRE has been shown to partially restore the central biological clock and maintain appetite regulation rhythms when feeding is restricted to a fixed daily phase, thereby improving metabolic disorders associated with chronic jet lag (Erren et al., 2022). Furthermore, in gut microbiome research, a recent review indicates that synchronizing eating with circadian rhythms, particularly through early TRE, can increase both the abundance and function of beneficial gut microbiota (Bajaj and Sharma, 2025). It also enhances gut barrier function and reduces systemic inflammation (Wang et al., 2023a).

Multiple studies in patients with T2DM suggest that TRE within an 8-10-h eating window can stabilize 24-h blood glucose fluctuations (Parr et al., 2023; Andriessen et al., 2022). Moreover, improvements in HbA1c have been sustained for up to one year, although a short-term 8-h TRE intervention did not significantly alter glucose levels (Bravo-Garcia et al., 2025). For individuals with nocturnal hyperglycemia, the clinical value of TRE may lie not only in reducing the eating window but also in aligning food intake with endogenous circadian metabolic regulation. To provide more specific guidance from a chrononutritional perspective for individuals with nocturnal hyperglycemia, early TRE is more effective than midday or late TRE in improving glycemic and anthropometric indicators. This suggests that patients with T2DM are advised to complete their dinner before 5 p.m. to more effectively improve blood glucose levels and restore insulin sensitivity, while avoiding late-night eating—a practice that may be more consistent with endogenous circadian metabolic regulation (Chen et al., 2026; Davis et al., 2022). Despite these potential benefits, TRE should be implemented cautiously in patients with T2DM, particularly in those receiving insulin, sulfonylureas, or meglitinides, because prolonged fasting or delayed meals may increase the risk of hypoglycemia. Before initiating TRE, patients should undergo an individualized risk assessment, medication review, and education on self-monitoring of blood glucose or continuous glucose monitoring and hypoglycemia management. Existing fasting-related diabetes guidance recommends medication adjustment and closer glucose monitoring during fasting periods, especially for patients using glucose-lowering agents with a high risk of hypoglycemia (Grajower and Horne, 2019; American Diabetes Association Professional Practice Committee for Diabetes, 2026). TRE may be unsuitable for patients with recurrent hypoglycemia, impaired hypoglycemia awareness, unstable glycemic control, pregnancy, active eating disorders, or severe comorbidities. Overall, for selected and clinically stable patients with T2DM, TRE may be considered as an adjunct dietary timing strategy under medical supervision.

### 4.3 Light intervention strategies

Light exposure is the most critical environmental factor regulating the central biological clock, transmitting light–dark cycle information through intrinsically photosensitive retinal ganglion cells (Komal et al., 2026). First, rhythmic management of the light environment is fundamental. With the rapid development of the economy and technological advancement, people’s daily lives have been profoundly transformed. Compared with several decades ago, human exposure to light at night has increased significantly. Artificial light at night (ALAN) refers to inappropriate exposure to illumination during nighttime hours, which disrupts normal physiological and behavioral processes (Walker et al., 2022). Research indicates that artificial light exposure at night triggers circadian disruption, directly reducing gut microbiota diversity and altering the abundance of pro-inflammatory bacteria (Lee et al., 2022); it also disturbs melatonin secretion and impairs insulin sensitivity (Salomon et al., 2025). Therefore, for individuals with T2DM, maintaining a dark bedroom environment and restoring natural light–dark cycles are recommended as primary measures to eliminate the risk of nocturnal hyperglycemia at the source (Li Q. et al., 2025). Furthermore, exposure to natural daylight during daytime working hours extends the duration of euglycemia in patients (Harmsen et al., 2026).

Second, photobiomodulation therapy (PBMT), a non-pharmacological intervention, exhibits unique potential in regulating gut microbiota. This modality utilizes non-ionizing light sources across the visible and infrared spectra to exert therapeutic effects (Anders et al., 2015). In mouse models, PBMT has been shown to improve insulin signaling pathways in adipose tissue and alleviate insulin resistance (Silva et al., 2018; Gong et al., 2020). The underlying mechanism is likely associated with the mitigation of mitochondrial dysfunction, reduction in pro-inflammatory cytokine production, and enhancement of antioxidant capacity. Notably, PBMT has also been shown to modulate the composition of the gut microbiota in healthy mice (Bicknell et al., 2019), suggesting its potential as a strategy to target the root causes of nocturnal glycemic instability. In clinical practice for patients with T2DM, PBMT has demonstrated significant benefits in lowering blood glucose and managing related complications (Wang et al., 2024). These findings provide novel research directions and practical insights for developing combined pharmacological and non-pharmacological intervention strategies for metabolic diseases such as T2DM. However, some studies have failed to observe significant therapeutic benefits (Lavery et al., 2008; Calbiague García et al., 2023), which may be attributed to the lack of standardized treatment parameters for PBMT—including frequency, dosage, and wavelength. Moreover, most current evidence is derived from animal studies, limiting its direct translatability to humans. Future studies should conduct more comprehensive evaluations of PBMT to provide more effective treatment strategies for T2DM patients.

## 5 Clinical translation challenges and future directions

Despite recent progress, the clinical translation of microbiota-based interventions for managing nocturnal hyperglycemia in T2DM still faces complex challenges arising from inter-individual variability and technical limitations.

First, current understanding of key microbial circadian features—such as the timing of metabolite peaks and the diurnal amplitude of beneficial or pathogenic bacterial abundance—is largely derived from animal studies. However, there are significant differences between the gut microbiota of animals and humans. For example, mice are nocturnal animals, with feeding, activity, and metabolic rhythms opposite to those of diurnal humans. Therefore, findings of murine studies should not be interpreted as direct temporal equivalents of human nocturnal metabolism. Rather, they provide mechanistic evidence that the host clock, the feeding-fasting cycle, the microbial oscillation of the gut and the microbial metabolites are functionally coupled (Leone et al., 2015; Altaha et al., 2022). Human studies provide complementary support rather than direct temporal confirmation. Arrhythmic gut microbiome signatures have been associated with T2DM risk in human cohorts (Reitmeier et al., 2020). Human glucose metabolism also shows circadian organization, including nocturnal changes in endogenous glucose production and altered fasting glucose rhythms in prediabetes and T2DM (Peng et al., 2022; Gubin et al., 2017). In addition, diurnal insulin sensitivity is altered in T2DM, indicating that human glucose regulation also deviates throughout the 24-h cycle (Lucidi et al., 2023). Human studies further show 24-h rhythms in circulating BAs, supporting the relevance of microbiota-related metabolic rhythms in human physiology (Al-Khaifi et al., 2018; Bello et al., 2024). Given that both humans and mice are vertebrates and share highly conserved core circadian clock pathways, the translational value of animal studies lies primarily in identifying conserved regulatory pathways, whereas human studies are required to determine the phase, magnitude, and clinical significance of these pathways in nocturnal glycemic control. Accurately characterizing circadian microbial profiles in humans is therefore essential to provide a scientific basis for chrono-nutritional strategies.

Another important limitation is that the causal relationship between microbiota dysrhythmia and nocturnal hyperglycemia in humans remains insufficiently established. Animal and microbiota-transfer studies provide mechanistic evidence that disrupted microbial rhythms can contribute to metabolic abnormalities (Thaiss et al., 2014; Altaha et al., 2022). In contrast, human studies to date mainly support associative or predictive relationships between gut microbiota alterations and glycemic dysregulation (Wang et al., 2022; Reitmeier et al., 2020). Therefore, microbiota dysrhythmia should be considered a potential contributor to nocturnal glycemic instability rather than a proven causal determinant in human T2DM. Future studies should combine 24-h longitudinal microbiome and metabolome sampling, continuous glucose monitoring, controlled diet timing, and causal-inference approaches to determine whether microbial rhythm disruption directly drives nocturnal hyperglycemia.

Second, several technical barriers impede clinical translation. Most current studies rely on single-time-point sampling, which fails to capture the dynamic fluctuations of gut microbial communities. Additionally, the gut microbiota is influenced by multiple factors, including genetics, diet, geography, and lifestyle, with medications such as metformin significantly altering microbial abundance (Pavlo et al., 2023). These limitations compromise the generalizability of observational evidence and complicate the interpretation of clinical trial outcomes. Future studies should enroll diverse populations with distinct dietary patterns, lifestyles, and geographic backgrounds, and establish cross-regional validation cohorts to assess the robustness of such interventions across different populations and settings.

At the intervention level, most existing probiotics are formulated as single strains or simple mixtures, which may not align with the highly individualized microbiota profiles of T2DM patients. Furthermore, unlike direct manipulation of host–microbiota rhythms, adjusting meal timing to align with the host circadian clock may offer a safer indirect approach. This approach leverages the host’s own circadian signals to reshape beneficial microbial rhythms, thereby helping to avoid the ecological risks associated with direct microbiota manipulation. However, the efficacy of such dietary timing interventions also depends on individual factors. Future efforts should focus on developing personalized, microbiota-based interventions guided by microbial enterotyping and on integrating dietary timing strategies adapted to different cultural contexts to ameliorate abnormal nocturnal glycemic fluctuations more effectively in T2DM patients.

## 6 Conclusion

This review systematically elucidates the mechanisms underlying nocturnal hyperglycemia in T2DM, centered on the “gut microbiota-circadian rhythm-metabolism axis**”**. Pathological features in patients with T2DM include alterations in gut microbial abundance and diversity, along with diminished circadian oscillations in microbiota activity, particularly characterized by the loss of rhythmic signaling during the nocturnal period. The core consequence is the disruption of the physiological circadian suppression of hepatic gluconeogenesis at night, which, in synergy with chronic low-grade inflammation triggered by intestinal barrier impairment, jointly drives nocturnal glycemic dysregulation. This indicates that nocturnal hyperglycemia in T2DM is not caused by a single factor but rather results from a systemic vicious cycle stemming from disrupted interactions between host and microbial circadian rhythms. In the future, with a deeper understanding of the precise mechanisms governing microbial and circadian regulation, a multidimensional therapeutic system may be established for patients with T2DM.

## Glossary

T2DM: Type 2 diabetes mellitus
SCFA: Short-chain fatty acid
BA: Bile acid
SCN: Suprachiasmatic nuclei
BMAL1: Brain and muscle arnt-like 1
CRY: Cryptochrome
PER: Period
EGP: Endogenous glucose production
GLP-1: Glucagon-like peptide-1
SBA: Secondary bile acid
BSH: Bile salt hydrolase
FXR: Farnesoid X receptor
TGR5: Takeda G protein-coupled receptor 5
LPS: Lipopolysaccharide
TNF-α: Tumor necrosis factor-α
IL-6: Interleukin-6
PI3K: Phosphatidylinositol 3-kinase
AKT: Protein kinase B
PEPCK: Phosphoenolpyruvate carboxykinase
HbA1c: Glycosylated hemoglobin A1c
MT: Melatonin
GLP-1R: GLP-1 receptor
TRE: Time-restricted eating
PBMT: Photobiomodulation therapy

## References

[ref1] Al-Khaifi A. Straniero S. Voronova V. Chernikova D. Sokolov V. Kumar C. . (2018). Asynchronous rhythms of circulating conjugated and unconjugated bile acids in the modulation of human metabolism. J. Intern. Med. 284, 546–559. doi: 10.1111/joim.12811, 29964306

[ref2] Alamili M. Bendtzen K. Lykkesfeldt J. Rosenberg J. Gögenur I. (2014). Pronounced inflammatory response to endotoxaemia during nighttime: a randomised cross-over trial. PLoS One 9:e87413. doi: 10.1371/journal.pone.0087413, 24475284 PMC3903723

[ref3] Alli S. R. Gorbovskaya I. Liu J. C. W. Kolla N. J. Brown L. Müller D. J. (2022). The Gut Microbiome in Depression and Potential Benefit of Prebiotics, Probiotics and Synbiotics: A Systematic Review of Clinical Trials and Observational Studies. Int. J. Mol. Sci. 23:4494. doi: 10.3390/ijms23094494, 35562885 PMC9101152

[ref4] Altaha B. Heddes M. Pilorz V. Niu Y. Gorbunova E. Gigl M. . (2022). Genetic and environmental circadian disruption induce weight gain through changes in the gut microbiome. Mol. Metab. 66:101628. doi: 10.1016/j.molmet.2022.101628, 36334897 PMC9672454

[ref5] American Diabetes Association Professional Practice Committee for Diabetes (2026). Facilitating Positive Health Behaviors and Well-being to Improve Health Outcomes: Standards of Care in Diabetes-2026. Diabetes Care 49, S89–s131. doi: 10.2337/dc26-S005Bajaj 41358898 PMC12690188

[ref6] Anders J. J. Lanzafame R. J. Arany P. R. (2015). Low-level light/laser therapy versus photobiomodulation therapy. Photomed. Laser Surg. 33, 183–184. doi: 10.1089/pho.2015.9848, 25844681 PMC4390214

[ref7] Ando H. Ushijima K. Fujimura A. (2013). Indirect effects of glucagon-like peptide-1 receptor agonist exendin-4 on the peripheral circadian clocks in mice. PLoS One 8:e81119. doi: 10.1371/journal.pone.0081119, 24260546 PMC3829942

[ref8] Andriessen C. Fealy C. E. Veelen A. van Beek S. M. M. Roumans K. H. M. Connell N. J. . (2022). Three weeks of time-restricted eating improves glucose homeostasis in adults with type 2 diabetes but does not improve insulin sensitivity: a randomised crossover trial. Diabetologia 65, 1710–1720. doi: 10.1007/s00125-022-05752-z, 35871650 PMC9477920

[ref9] Bajaj P. Sharma M. (2025). Chrononutrition and gut health: exploring the relationship between meal timing and the gut microbiome. Curr. Nutr. Rep. 14:79:79. doi: 10.1007/s13668-025-00670-z, 40488812

[ref10] Beli E. Prabakaran S. Krishnan P. Evans-Molina C. Grant M. B. (2019). Loss of Diurnal Oscillatory Rhythms in Gut Microbiota Correlates with Changes in Circulating Metabolites in Type 2 Diabetic db/db Mice. Nutrients 11:2310. doi: 10.3390/nu11102310, 31569518 PMC6835667

[ref11] Bello A. T. Sarafian M. H. Wimborne E. A. Middleton B. Revell V. L. Raynaud F. I. . (2024). Exposing 24-hour cycles in bile acids of male humans. Nat. Commun. 15:10014. doi: 10.1038/s41467-024-53673-9, 39562795 PMC11576969

[ref12] Bicknell B. Liebert A. Johnstone D. Kiat H. (2019). Photobiomodulation of the microbiome: implications for metabolic and inflammatory diseases. Lasers Med. Sci. 34, 317–327. doi: 10.1007/s10103-018-2594-6, 30074108

[ref13] Bishehsari F. Post Z. Swanson G. R. Keshavarzian A. (2025). Circadian Rhythms in Gastroenterology: The Biological Clock's Impact on Gut Health. Gastroenterology 169, 1380–1396. doi: 10.1053/j.gastro.2025.06.017, 40588189 PMC13552429

[ref14] Borgmann D. Fenselau H. (2024). Vagal pathways for systemic regulation of glucose metabolism. Semin. Cell Dev. Biol. 156, 244–252. doi: 10.1016/j.semcdb.2023.07.010, 37500301

[ref15] Boughton C. K. Daly A. Thabit H. Hartnell S. Herzig D. Vogt A. . (2021). Day-to-day variability of insulin requirements in the inpatient setting: Observations during fully closed-loop insulin delivery. Diabetes Obes. Metab. 23, 1978–1982. doi: 10.1111/dom.14396, 33822461

[ref16] Bravo-Garcia A. P. Radford B. E. Hall R. C. Broome S. C. Tee N. Arthur B. . (2025). Combined effects of time-restricted eating and exercise on short-term blood glucose management in individuals with Type 2 Diabetes Mellitus: The TREx study, a randomised controlled trial. Diabetes Res. Clin. Pract. 222:112081. doi: 10.1016/j.diabres.2025.112081, 40064299

[ref17] Brignardello J. Fountana S. Posma J. M. Chambers E. S. Nicholson J. K. Wist J. . (2022). Characterization of diet-dependent temporal changes in circulating short-chain fatty acid concentrations: A randomized crossover dietary trial. Am. J. Clin. Nutr. 116, 1368–1378. doi: 10.1093/ajcn/nqab211, 36137188 PMC9630877

[ref18] Cai J. Peng P. Lu J. Shen Y. Wang C. Mo Y. . (2024). Severe dawn phenomenon predicts long-term risk of all-cause mortality in patients with type 2 diabetes. Diabetes Metab. Res. Rev. 40:e3813. doi: 10.1002/dmrr.3813, 38767128

[ref19] Calbiague García V. Cadiz B. Herrera P. Díaz A. Schmachtenberg O. (2023). Evaluation of Photobiomodulation and Boldine as Alternative Treatment Options in Two Diabetic Retinopathy Models. Int. J. Mol. Sci. 24:7918. doi: 10.3390/ijms24097918, 37175628 PMC10178531

[ref20] Cani P. D. Amar J. Iglesias M. A. Poggi M. Knauf C. Bastelica D. . (2007). Metabolic endotoxemia initiates obesity and insulin resistance. Diabetes 56, 1761–1772. doi: 10.2337/db06-1491, 17456850

[ref21] Carrizales-Sánchez A. K. Tamez-Rivera O. Rodríguez-Gutiérrez N. A. Elizondo-Montemayor L. Gradilla-Hernández M. S. García-Rivas G. . (2023). Characterization of gut microbiota associated with metabolic syndrome and type-2 diabetes mellitus in Mexican pediatric subjects. BMC Pediatr. 23:210. doi: 10.1186/s12887-023-03983-6, 37138212 PMC10155456

[ref22] Chen M. Lin Y. Dang Y. Xiao Y. Zhang F. Sun G. . (2023). Reprogramming of rhythmic liver metabolism by intestinal clock. J. Hepatol. 79, 741–757. doi: 10.1016/j.jhep.2023.04.040, 37230230

[ref23] Chen Y. Cui W. Li X. Yang H. (2021). Interaction Between Commensal Bacteria, Immune Response and the Intestinal Barrier in Inflammatory Bowel Disease. Front. Immunol. 12:761981. doi: 10.3389/fimmu.2021.761981, 34858414 PMC8632219

[ref24] Chen Y. E. Tsai H. L. Tu Y. K. Chen L. W. (2026). Effects of timing and eating duration of time restricted eating on metabolic outcomes: systematic review and network meta-analysis. BMJ Med. 5:e001071. doi: 10.1136/bmjmed-2024-001071, 41586347 PMC12829361

[ref25] Cheng W. Y. Lam K. L. Pik-Shan Kong A. Chi-Keung Cheung P. (2020). Prebiotic supplementation (beta-glucan and inulin) attenuates circadian misalignment induced by shifted light-dark cycle in mice by modulating circadian gene expression. Food Res. Int. 137:109437:109437. doi: 10.1016/j.foodres.2020.109437, 33233118

[ref26] Choi H. Rao M. C. Chang E. B. (2021). Gut microbiota as a transducer of dietary cues to regulate host circadian rhythms and metabolism. Nat. Rev. Gastroenterol. Hepatol. 18, 679–689. doi: 10.1038/s41575-021-00452-2, 34002082 PMC8521648

[ref27] Civelek E. Ozturk Civelek D. Akyel Y. K. Kaleli Durman D. Okyar A. (2023). Circadian dysfunction in adipose tissue: chronotherapy in metabolic diseases. Biology 12:1077. doi: 10.3390/biology12081077, 37626963 PMC10452180

[ref28] Creely S. J. McTernan P. G. Kusminski C. M. Fisher f M. Da Silva N. F. Khanolkar M. . (2007). Lipopolysaccharide activates an innate immune system response in human adipose tissue in obesity and type 2 diabetes. Am. J. Physiol. Endocrinol. Metab. 292, E740–E747. doi: 10.1152/ajpendo.00302.2006, 17090751

[ref29] Crudele L. Gadaleta R. M. Cariello M. Moschetta A. (2023). Gut microbiota in the pathogenesis and therapeutic approaches of diabetes. EBioMedicine 97:104821. doi: 10.1016/j.ebiom.2023.104821, 37804567 PMC10570704

[ref30] Cui J. Ramesh G. Wu M. Jensen E. T. Crago O. Bertoni A. G. . (2022). Butyrate-producing bacteria and insulin homeostasis: the microbiome and insulin longitudinal evaluation study (MILES). Diabetes 71, 2438–2446. doi: 10.2337/db22-0168, 35972231 PMC9630078

[ref31] Curtis A. M. Fagundes C. T. Yang G. Palsson-McDermott E. M. Wochal P. McGettrick A. F. . (2015). Circadian control of innate immunity in macrophages by miR-155 targeting Bmal1. Proc. Natl. Acad. Sci. USA 112, 7231–7236. doi: 10.1073/pnas.1501327112, 25995365 PMC4466714

[ref32] Dantas Machado A. C. Brown S. D. Lingaraju A. Sivaganesh V. Martino C. Chaix A. . (2022). Diet and feeding pattern modulate diurnal dynamics of the ileal microbiome and transcriptome. Cell Rep. 40:111008. doi: 10.1016/j.celrep.2022.111008, 35793637 PMC9296000

[ref33] Davis R. Rogers M. Coates A. M. Leung G. K. W. Bonham M. P. (2022). The impact of meal timing on risk of weight gain and development of obesity: a review of the current evidence and opportunities for dietary intervention. Curr. Diab. Rep. 22, 147–155. doi: 10.1007/s11892-022-01457-0, 35403984 PMC9010393

[ref34] Delpino F. M. Figueiredo L. M. Nunes B. P. (2021). Effects of melatonin supplementation on diabetes: A systematic review and meta-analysis of randomized clinical trials. Clin. Nutr. 40, 4595–4605. doi: 10.1016/j.clnu.2021.06.007, 34229264

[ref35] Desmet L. Thijs T. Segers A. Verbeke K. Depoortere I. (2021). Chronodisruption by chronic jetlag impacts metabolic and gastrointestinal homeostasis in male mice. Acta Physiol 233:e13703. doi: 10.1111/apha.13703, 34107165

[ref36] Ding G. Li X. Hou X. Zhou W. Gong Y. Liu F. . (2021). REV-ERB in GABAergic neurons controls diurnal hepatic insulin sensitivity. Nature 592, 763–767. doi: 10.1038/s41586-021-03358-w, 33762728 PMC8085086

[ref37] Ding L. Xiao X. H. (2020). Gut microbiota: closely tied to the regulation of circadian clock in the development of type 2 diabetes mellitus. Chin. Med. J. 133, 817–825. doi: 10.1097/cm9.0000000000000702, 32106122 PMC7147650

[ref38] Du W. Jiang S. Yin S. Wang R. Zhang C. Yin B. C. . (2024). The microbiota-dependent tryptophan metabolite alleviates high-fat diet-induced insulin resistance through the hepatic AhR/TSC2/mTORC1 axis. Proc. Natl. Acad. Sci. USA 121:e2400385121. doi: 10.1073/pnas.2400385121, 39167602 PMC11363250

[ref39] Erren T. C. Piekarski C. Reiter R. J. (2022). Chronodisruption: origin, roots, and developments of an 18-year-old concept. Comment on Desmet et al. Time-restricted feeding in mice prevents the disruption of the peripheral circadian clocks and its metabolic impact during chronic jetlag. Nutrients 14:315. doi: 10.3390/nu14020315, 35057496 PMC8779712

[ref40] Forslund K. Hildebrand F. Nielsen T. Falony G. Le Chatelier E. Sunagawa S. . (2015). Disentangling type 2 diabetes and metformin treatment signatures in the human gut microbiota. Nature 528, 262–266. doi: 10.1038/nature15766, 26633628 PMC4681099

[ref41] Frazier K. Chang E. B. (2020). Intersection of the Gut Microbiome and Circadian Rhythms in Metabolism. Trends Endocrinol. Metab. 31, 25–36. doi: 10.1016/j.tem.2019.08.013, 31677970 PMC7308175

[ref42] Frazier K. Manzoor S. Carroll K. DeLeon O. Miyoshi S. Miyoshi J. . (2023). Gut microbes and the liver circadian clock partition glucose and lipid metabolism. J. Clin. Invest. 133:e162515. doi: 10.1172/jci162515, 37712426 PMC10503806

[ref43] Garaulet M. Qian J. Florez J. C. Arendt J. Saxena R. Scheer F. (2020). Melatonin Effects on Glucose Metabolism: Time To Unlock the Controversy. Trends Endocrinol. Metab. 31, 192–204. doi: 10.1016/j.tem.2019.11.011, 31901302 PMC7349733

[ref44] Gioiello A. Rosatelli E. Cerra B. (2024). Patented Farnesoid X receptor modulators: a review (2019 - present). Expert Opin. Ther. Pat. 34, 547–564. doi: 10.1080/13543776.2024.2314296, 38308658

[ref45] Gong L. Zou Z. Huang L. Guo S. Xing D. (2020). Photobiomodulation therapy decreases free fatty acid generation and release in adipocytes to ameliorate insulin resistance in type 2 diabetes. Cell. Signal. 67:109491:109491. doi: 10.1016/j.cellsig.2019.109491, 31809873

[ref46] Govindarajan K. MacSharry J. Casey P. G. Shanahan F. Joyce S. A. Gahan C. G. (2016). Unconjugated bile acids influence expression of circadian genes: a potential mechanism for microbe-host crosstalk. PLoS One 11:e0167319. doi: 10.1371/journal.pone.0167319, 27907092 PMC5132238

[ref47] Grajower M. M. Horne B. D. (2019). Clinical Management of Intermittent Fasting in Patients with Diabetes Mellitus. Nutrients 11:873. doi: 10.3390/nu11040873, 31003482 PMC6521152

[ref48] Gubin D. G. Nelaeva A. A. Uzhakova A. E. Hasanova Y. V. Cornelissen G. Weinert D. (2017). Disrupted circadian rhythms of body temperature, heart rate and fasting blood glucose in prediabetes and type 2 diabetes mellitus. Chronobiol. Int. 34, 1136–1148. doi: 10.1080/07420528.2017.1347670, 28759269

[ref49] Guo Q. Wang X. Ke J. Hou X. Shen G. Li S. . (2025). Chayote pectin regulates blood glucose through the gut-liver axis: Gut microbes/SCFAs/FoxO1 signaling pathways. Food Res. Int. 202:115706. doi: 10.1016/j.foodres.2025.115706, 39967162

[ref50] Han X. Chen Y. Wang L. Shen Y. Zhang X. Chen Q. (2025). Research Progress of Neurohormone Signaling Pathway in Diabetes Based on Brain-Gut-Pancreas Axis. J. Shaanxi Univ. Chin. Med. 48, 136–141. doi: 10.13424/j.cnki.jsctcm.2025.03.023

[ref51] Harmsen J. F. Habets I. Biancolin A. D. Lesniewska A. Phillips N. E. Metz L. . (2026). Natural daylight during office hours improves glucose control and whole-body substrate metabolism. Cell Metab. 38, 65–81. doi: 10.1016/j.cmet.2025.11.006, 41418772

[ref52] He Q. L. Wang H. C. Ma Y. K. Yang R. L. Dai Z. F. Yang J. N. . (2023). Changes in the microbiota and their roles in patients with type 2 diabetes mellitus. Curr. Microbiol. 80:132. doi: 10.1007/s00284-023-03219-x, 36894807

[ref53] Holst J. J. Andersen D. B. Grunddal K. V. (2022). Actions of glucagon-like peptide-1 receptor ligands in the gut. Br. J. Pharmacol. 179, 727–742. doi: 10.1111/bph.15611, 34235727 PMC8820219

[ref54] Hotamisligil G. S. (2006). Inflammation and metabolic disorders. Nature 444, 860–867. doi: 10.1038/nature05485, 17167474

[ref55] Iesanu M. I. Zahiu C. D. M. Dogaru I. A. Chitimus D. M. Pircalabioru G. G. Voiculescu S. E. . (2022). Melatonin-microbiome two-sided interaction in dysbiosis-associated conditions. Antioxidants 11:2244. doi: 10.3390/antiox11112244, 36421432 PMC9686962

[ref56] Kabahizi A. Wallace B. Lieu L. Chau D. Dong Y. Hwang E. S. . (2022). Glucagon-like peptide-1 (GLP-1) signalling in the brain: From neural circuits and metabolism to therapeutics. Br. J. Pharmacol. 179, 600–624. doi: 10.1111/bph.15682, 34519026 PMC8820188

[ref57] Kalkan A. T. Yorulmaz G. Akalin A. Dinleyici E. C. (2025). Intestinal microbiota composition in patients with type 2 diabetes and effects of oral antidiabetics. J. Clin. Med. 14:786. doi: 10.3390/jcm14082786, 40283615 PMC12027695

[ref58] Karamitri A. Jockers R. (2019). Melatonin in type 2 diabetes mellitus and obesity. Nat. Rev. Endocrinol. 15, 105–125. doi: 10.1038/s41574-018-0130-1, 30531911

[ref59] Kavakli I. H. Ozturk N. Baris I. (2022). Protein interaction networks of the mammalian core clock proteins. Adv. Protein Chem. Struct. Biol. 131, 207–233. doi: 10.1016/bs.apcsb.2022.04.001, 35871891

[ref60] Kim K. S. Park J. S. Hwang E. Park M. J. Shin H. Y. Lee Y. H. . (2024). GLP-1 increases preingestive satiation via hypothalamic circuits in mice and humans. Science 385, 438–446. doi: 10.1126/science.adj2537, 38935778 PMC11961025

[ref61] Komal R. Beier C. Nath A. Grimes W. N. Wang H. Berry M. . (2026). ipRGC properties prevent light from shifting the SCN clock during daytime. Nature 650, 942–950. doi: 10.1038/s41586-025-09894-z, 41501453

[ref62] Kornum D. S. Krogh K. Keller J. Malagelada C. Drewes A. M. Brock C. (2025). Diabetic gastroenteropathy: a pan-alimentary complication. Diabetologia 68, 905–919. doi: 10.1007/s00125-025-06365-y, 39934370 PMC12021976

[ref63] Kyoko O. O. Kono H. Ishimaru K. Miyake K. Kubota T. Ogawa H. . (2014). Expressions of tight junction proteins Occludin and Claudin-1 are under the circadian control in the mouse large intestine: implications in intestinal permeability and susceptibility to colitis. PLoS One 9:e98016. doi: 10.1371/journal.pone.0098016, 24845399 PMC4028230

[ref64] Lauritzen E. S. Kampmann U. Pedersen M. G. B. Christensen L. L. Jessen N. Møller N. . (2022). Three months of melatonin treatment reduces insulin sensitivity in patients with type 2 diabetes-A randomized placebo-controlled crossover trial. J. Pineal Res. 73:e12809. doi: 10.1111/jpi.12809, 35619221 PMC9540532

[ref65] Lavery L. A. Murdoch D. P. Williams J. Lavery D. C. (2008). Does anodyne light therapy improve peripheral neuropathy in diabetes? A double-blind, sham-controlled, randomized trial to evaluate monochromatic infrared photoenergy. Diabetes Care 31, 316–321. doi: 10.2337/dc07-1794, 17977931

[ref66] Lee C. C. Liang F. Lee I. C. Lu T. H. Shan Y. Y. Jeng C. F. . (2022). External light-dark cycle shapes gut microbiota through intrinsically photosensitive retinal ganglion cells. EMBO Rep. 23:e52316. doi: 10.15252/embr.202052316, 35476894 PMC9171413

[ref67] Leone V. Gibbons S. M. Martinez K. Hutchison A. L. Huang E. Y. Cham C. M. . (2015). Effects of diurnal variation of gut microbes and high-fat feeding on host circadian clock function and metabolism. Cell Host Microbe 17, 681–689. doi: 10.1016/j.chom.2015.03.006, 25891358 PMC4433408

[ref68] Li J. Yu K. Sui X. Deng H. Leng Y. Liu T. (2025). Gut jet lag: how circadian rhythm disruption undermines the Chrono-Microbiota-Motility axis and induces functional constipation. Front. Nutr. 12:1678482. doi: 10.3389/fnut.2025.1678482, 41112730 PMC12528175

[ref69] Li Q. Xu Y. X. Lu X. Z. Shen Y. T. Wan Y. H. Su P. Y. . (2025). Impact of bedroom light exposure on glucose metabolic markers and the role of circadian-dependent meal timing: A population-based cross-sectional study. Ecotoxicol. Environ. Saf. 290:117589. doi: 10.1016/j.ecoenv.2024.117589, 39729942

[ref70] Li W. Wang Z. Cao J. Dong Y. Chen Y. (2023). Melatonin improves the homeostasis of mice gut microbiota rhythm caused by sleep restriction. Microbes Infect. 25:105121. doi: 10.1016/j.micinf.2023.105121, 36804006

[ref71] Li X. Ding K. Ma Y. Xiao H. Liu Y. Wu G. . (2025). Gut microbial signatures in type 2 diabetes are highly associated with geographic region, diet habits and common comorbidities: Insights from a bioinformatics analysis of Chinese population. Diabetes Res. Clin. Pract. 229:112438. doi: 10.1016/j.diabres.2025.112438, 40902902

[ref72] Li Y. Liu T. Qin L. Wu L. (2023). Effects of probiotic administration on overweight or obese children: a meta-analysis and systematic review. J. Transl. Med. 21:525. doi: 10.1186/s12967-023-04319-9, 37542325 PMC10401801

[ref73] Litichevskiy L. Thaiss C. A. (2022). The Oscillating Gut Microbiome and Its Effects on Host Circadian Biology. Annu. Rev. Nutr. 42, 145–164. doi: 10.1146/annurev-nutr-062320-111321, 35576592

[ref74] Liu B. Fan L. Wang Y. Wang H. Yan Y. Chen S. . (2024). Gut microbiota regulates host melatonin production through epithelial cell MyD88. Gut Microbes 16:2313769. doi: 10.1080/19490976.2024.2313769, 38353638 PMC10868534

[ref75] Lucidi P. Perriello G. Porcellati F. Pampanelli S. De Fano M. Tura A. . (2023). Diurnal cycling of insulin sensitivity in type 2 diabetes: evidence for deviation from physiology at an early stage. Diabetes 72, 1364–1373. doi: 10.2337/db22-0721, 37440717 PMC10866740

[ref76] Luo W. Yin Z. Zhang M. Huang X. Yin J. (2024). Dietary *Lactobacillus delbrueckii* Affects Ileal Bacterial Composition and Circadian Rhythms in Pigs. Animals 14:412. doi: 10.3390/ani14030412, 38338054 PMC10854795

[ref77] Martchenko S. E. Martchenko A. Biancolin A. D. Waller A. Brubaker P. L. (2021). L-cell Arntl is required for rhythmic glucagon-like peptide-1 secretion and maintenance of intestinal homeostasis. Mol. Metab. 54:101340. doi: 10.1016/j.molmet.2021.101340, 34520858 PMC8489154

[ref78] Martchenko S. E. Martchenko A. Cox B. J. Naismith K. Waller A. Gurges P. . (2020). Circadian GLP-1 Secretion in Mice Is Dependent on the Intestinal Microbiome for Maintenance of Diurnal Metabolic Homeostasis. Diabetes 69, 2589–2602. doi: 10.2337/db20-0262, 32928871

[ref79] McMullan C. J. Schernhammer E. S. Rimm E. B. Hu F. B. Forman J. P. (2013). Melatonin secretion and the incidence of type 2 diabetes. JAMA 309, 1388–1396. doi: 10.1001/jama.2013.2710, 23549584 PMC3804914

[ref80] Mei Z. Wang F. Bhosle A. Dong D. Mehta R. Ghazi A. . (2024). Strain-specific gut microbial signatures in type 2 diabetes identified in a cross-cohort analysis of 8,117 metagenomes. Nat. Med. 30, 2265–2276. doi: 10.1038/s41591-024-03067-7, 38918632 PMC11620793

[ref81] Meshkani R. Adeli K. (2009). Hepatic insulin resistance, metabolic syndrome and cardiovascular disease. Clin. Biochem. 42, 1331–1346. doi: 10.1016/j.clinbiochem.2009.05.018, 19501581

[ref82] Nogueira T. C. Lellis-Santos C. Jesus D. S. Taneda M. Rodrigues S. C. Amaral F. G. . (2011). Absence of melatonin induces night-time hepatic insulin resistance and increased gluconeogenesis due to stimulation of nocturnal unfolded protein response. Endocrinology 152, 1253–1263. doi: 10.1210/en.2010-1088, 21303940

[ref83] Park J. S. Kim K. S. Choi H. J. (2025). Glucagon-Like Peptide-1 and Hypothalamic Regulation of Satiation: Cognitive and Neural Insights from Human and Animal Studies. Diabetes Metab. J. 49, 333–347. doi: 10.4093/dmj.2025.0106, 40367985 PMC12086555

[ref84] Parr E. B. Steventon-Lorenzen N. Johnston R. Maniar N. Devlin B. L. Lim K. H. C. . (2023). Time-restricted eating improves measures of daily glycaemic control in people with type 2 diabetes. Diabetes Res. Clin. Pract. 197:110569. doi: 10.1016/j.diabres.2023.110569, 36738837

[ref85] Pavlo P. Kamyshna I. Kamyshnyi A. (2023). Effects of metformin on the gut microbiota: A systematic review. Mol. Metab. 77:101805. doi: 10.1016/j.molmet.2023.101805, 37696355 PMC10518565

[ref86] Peng F. Li X. Xiao F. Zhao R. Sun Z. (2022). Circadian clock, diurnal glucose metabolic rhythm, and dawn phenomenon. Trends Neurosci. 45, 471–482. doi: 10.1016/j.tins.2022.03.010, 35466006 PMC9117496

[ref87] Perry R. J. Camporez J. G. Kursawe R. Titchenell P. M. Zhang D. Perry C. J. . (2015). Hepatic acetyl CoA links adipose tissue inflammation to hepatic insulin resistance and type 2 diabetes. Cell 160, 745–758. doi: 10.1016/j.cell.2015.01.012, 25662011 PMC4498261

[ref88] Picinato M. C. Hirata A. E. Cipolla-Neto J. Curi R. Carvalho C. R. Anhê G. F. . (2008). Activation of insulin and IGF-1 signaling pathways by melatonin through MT1 receptor in isolated rat pancreatic islets. J. Pineal Res. 44, 88–94. doi: 10.1111/j.1600-079X.2007.00493.x, 18078453

[ref89] Qian J. Morris C. J. Phillips A. J. K. Li P. Rahman S. A. Wang W. . (2022). Unanticipated daytime melatonin secretion on a simulated night shift schedule generates a distinctive 24-h melatonin rhythm with antiphasic daytime and nighttime peaks. J. Pineal Res. 72:e12791. doi: 10.1111/jpi.12791, 35133678 PMC8930611

[ref90] Rahman A. A. Stavely R. Pan W. Ott L. Ohishi K. Ohkura T. . (2024). Optogenetic Activation of Cholinergic Enteric Neurons Reduces Inflammation in Experimental Colitis. Cell. Mol. Gastroenterol. Hepatol. 17, 907–921. doi: 10.1016/j.jcmgh.2024.01.012, 38272444 PMC11026705

[ref91] Razavi S. Amirmozafari N. Zahedi Bialvaei A. Navab-Moghadam F. Khamseh M. E. Alaei-Shahmiri F. . (2024). Gut microbiota composition and type 2 diabetes: are these subjects linked together? Heliyon 10:e39464. doi: 10.1016/j.heliyon.2024.e39464, 39469674 PMC11513563

[ref92] Reitmeier S. Kiessling S. Clavel T. List M. Almeida E. L. Ghosh T. S. . (2020). Arrhythmic gut microbiome signatures predict risk of type 2 diabetes. Cell Host Microbe 28, 258–272.e6. doi: 10.1016/j.chom.2020.06.004 32619440

[ref93] Rinaldi E. van der Heide F. C. Bonora E. Trombetta M. Zusi C. Kroon A. A. . (2023). Lower heart rate variability, an index of worse autonomic function, is associated with worse beta cell response to a glycemic load in vivo-The Maastricht Study. Cardiovasc. Diabetol. 22:105. doi: 10.1186/s12933-023-01837-0, 37143089 PMC10161476

[ref94] Rosli N. S. A. Abd Gani S. Khayat M. E. Zaidan U. H. Ismail A. Abdul Rahim M. B. H. (2023). Short-chain fatty acids: possible regulators of insulin secretion. Mol. Cell. Biochem. 478, 517–530. doi: 10.1007/s11010-022-04528-8, 35943655

[ref95] Rubio-Sastre P. Scheer F. A. Gómez-Abellán P. Madrid J. A. Garaulet M. (2014). Acute melatonin administration in humans impairs glucose tolerance in both the morning and evening. Sleep 37, 1715–1719. doi: 10.5665/sleep.4088, 25197811 PMC4173928

[ref96] Russell D. W. (2003). The enzymes, regulation, and genetics of bile acid synthesis. Annu. Rev. Biochem. 72, 137–174. doi: 10.1146/annurev.biochem.72.121801.161712, 12543708

[ref97] Salehi M. Gastaldelli A. D'Alessio D. A. (2014). Blockade of glucagon-like peptide 1 receptor corrects postprandial hypoglycemia after gastric bypass. Gastroenterology 146, 669–680.e2. doi: 10.1053/j.gastro.2013.11.044, 24315990 PMC3943944

[ref98] Salomon I. Sam S. Rehman Y. U. Hope I. M. (2025). Artificial light exposure at night: A hidden risk factor for type 2 diabetes. Sleep Med. X 10:100146. doi: 10.1016/j.sleepx.2025.100146, 40606287 PMC12221600

[ref99] Secher A. Jelsing J. Baquero A. F. Hecksher-Sørensen J. Cowley M. A. Dalbøge L. S. . (2014). The arcuate nucleus mediates GLP-1 receptor agonist liraglutide-dependent weight loss. J. Clin. Invest. 124, 4473–4488. doi: 10.1172/jci75276, 25202980 PMC4215190

[ref100] Segers A. Desmet L. Thijs T. Verbeke K. Tack J. Depoortere I. (2019). The circadian clock regulates the diurnal levels of microbial short-chain fatty acids and their rhythmic effects on colon contractility in mice. Acta Physiol (Oxf.) 225:e13193. doi: 10.1111/apha.13193, 30269420

[ref101] Sharma S. Singh H. Ahmad N. Mishra P. Tiwari A. (2015). The role of melatonin in diabetes: therapeutic implications. Arch. Endocrinol. Metab. 59, 391–399. doi: 10.1590/2359-3997000000098, 26331226

[ref102] Sharma S. A. Oladejo S. O. Kuang Z. (2025). Chemical interplay between gut microbiota and epigenetics: Implications in circadian biology. Cell Chem. Biol. 32, 61–82. doi: 10.1016/j.chembiol.2024.04.016, 38776923 PMC11569273

[ref103] Shi L. Jin L. Huang W. (2023). Bile Acids, Intestinal Barrier Dysfunction, and Related Diseases. Cells 12:1888. doi: 10.3390/cells12141888, 37508557 PMC10377837

[ref104] Siemian J. N. Arenivar M. A. Sarsfield S. Aponte Y. (2021). Hypothalamic control of interoceptive hunger. Curr. Biol. 31, 3797–3809.e5. doi: 10.1016/j.cub.2021.06.048, 34273280 PMC8440483

[ref105] Silva G. Ferraresi C. de Almeida R. T. Motta M. L. Paixão T. Ottone V. O. . (2018). Infrared photobiomodulation (PBM) therapy improves glucose metabolism and intracellular insulin pathway in adipose tissue of high-fat fed mice. Lasers Med. Sci. 33, 559–571. doi: 10.1007/s10103-017-2408-2, 29247431

[ref106] Siptroth J. Moskalenko O. Krumbiegel C. Ackermann J. Koch I. Pospisil H. (2023). Variation of butyrate production in the gut microbiome in type 2 diabetes patients. Int. Microbiol. 26, 601–610. doi: 10.1007/s10123-023-00324-6, 36780038 PMC10397123

[ref107] Snelson M. de Pasquale C. Ekinci E. I. Coughlan M. T. (2021). Gut microbiome, prebiotics, intestinal permeability and diabetes complications. Best Pract. Res. Clin. Endocrinol. Metab. 35:101507. doi: 10.1016/j.beem.2021.101507, 33642218

[ref108] Sroka-Oleksiak A. Młodzińska A. Bulanda M. Salamon D. Major P. Stanek M. . (2020). Metagenomic Analysis of Duodenal Microbiota Reveals a Potential Biomarker of Dysbiosis in the Course of Obesity and Type 2 Diabetes: A Pilot Study. J. Clin. Med. 9:369. doi: 10.3390/jcm9020369, 32013181 PMC7074165

[ref109] Stožer A. Paradiž Leitgeb E. Pohorec V. Dolenšek J. Križančić Bombek L. Gosak M. . (2021). The Role of cAMP in Beta Cell Stimulus-Secretion and Intercellular Coupling. Cells 10:1658. doi: 10.3390/cells10071658, 34359828 PMC8304079

[ref110] Sun Q. Ho C. T. Zhang X. Liu Y. Zhang R. Wu Z. (2022). Strategies for circadian rhythm disturbances and related psychiatric disorders: a new cue based on plant polysaccharides and intestinal microbiota. Food Funct. 13, 1048–1061. doi: 10.1039/d1fo02716f, 35050270

[ref111] Tahara Y. Yamazaki M. Sukigara H. Motohashi H. Sasaki H. Miyakawa H. . (2018). Gut Microbiota-Derived Short Chain Fatty Acids Induce Circadian Clock Entrainment in Mouse Peripheral Tissue. Sci. Rep. 8:1395. doi: 10.1038/s41598-018-19836-7, 29362450 PMC5780501

[ref112] Tan J. Taitz J. J. Ni D. Potier-Villette C. Pinget G. Pulpitel T. . (2025). Gut microbiota-derived extracellular vesicles exhibit diurnal regulation and activate hepatic gluconeogenesis. Mol. Metab. 98:102180. doi: 10.1016/j.molmet.2025.102180, 40484172 PMC12221565

[ref113] Tawulie D. Jin L. Shang X. Li Y. Sun L. Xie H. . (2023). Jiang-Tang-San-Huang pill alleviates type 2 diabetes mellitus through modulating the gut microbiota and bile acids metabolism. Phytomedicine 113:154733. doi: 10.1016/j.phymed.2023.154733, 36870307

[ref114] Thaiss C. A. Levy M. Korem T. Dohnalová L. Shapiro H. Jaitin D. A. . (2016). Microbiota diurnal rhythmicity programs host transcriptome oscillations. Cell 167, 1495–1510.e12. doi: 10.1016/j.cell.2016.11.003 27912059

[ref115] Thaiss C. A. Zeevi D. Levy M. Zilberman-Schapira G. Suez J. Tengeler A. C. . (2014). Transkingdom control of microbiota diurnal oscillations promotes metabolic homeostasis. Cell 159, 514–529. doi: 10.1016/j.cell.2014.09.048, 25417104

[ref116] Tilg H. Moschen A. R. (2008). Inflammatory mechanisms in the regulation of insulin resistance. Mol. Med. 14, 222–231. doi: 10.2119/2007-00119.Tilg, 18235842 PMC2215762

[ref117] Tordjman S. Chokron S. Delorme R. Charrier A. Bellissant E. Jaafari N. . (2017). Melatonin: Pharmacology, Functions and Therapeutic Benefits. Curr. Neuropharmacol. 15, 434–443. doi: 10.2174/1570159x14666161228122115, 28503116 PMC5405617

[ref118] Toshimitsu T. Irie J. (2025). An update and overview of the various health-related benefits of probiotics: A focus on clinical trials demonstrating efficacy, tolerability and use in patients with impaired glucose tolerance and type 2 diabetes. Diabetes Obes. Metab. 27, 15–22. doi: 10.1111/dom.16273, 39989436 PMC11894779

[ref119] Tschöp M. Nogueiras R. Ahrén B. (2023). Gut hormone-based pharmacology: novel formulations and future possibilities for metabolic disease therapy. Diabetologia 66, 1796–1808. doi: 10.1007/s00125-023-05929-0, 37209227 PMC10474213

[ref120] Tuomi T. Nagorny C. L. F. Singh P. Bennet H. Yu Q. Alenkvist I. . (2016). Increased Melatonin Signaling Is a Risk Factor for Type 2 Diabetes. Cell Metab. 23, 1067–1077. doi: 10.1016/j.cmet.2016.04.009, 27185156

[ref121] Walker W. H. n. Bumgarner J. R. Becker-Krail D. D. May L. E. Liu J. A. Nelson R. J. (2022). Light at night disrupts biological clocks, calendars, and immune function. Semin. Immunopathol. 44, 165–173. doi: 10.1007/s00281-021-00899-0, 34731290 PMC8564795

[ref122] Wang H. Gou W. Su C. Du W. Zhang J. Miao Z. . (2022). Association of gut microbiota with glycaemic traits and incident type 2 diabetes, and modulation by habitual diet: a population-based longitudinal cohort study in Chinese adults. Diabetologia 65, 1145–1156. doi: 10.1007/s00125-022-05687-5, 35357559 PMC9174105

[ref123] Wang H. Li Q. Xu R. Su Y. Zhu W. (2023a). Time-restricted feeding affects colonic nutrient substrates and modulates the diurnal fluctuation of microbiota in pigs. Front. Microbiol. 14:1162482. doi: 10.3389/fmicb.2023.1162482, 37275162 PMC10235616

[ref124] Wang H. Xu R. Li Q. Su Y. Zhu W. (2023b). Daily fluctuation of colonic microbiome in response to nutrient substrates in a pig model. NPJ Biofilms Microbiomes 9:85. doi: 10.1038/s41522-023-00453-w, 37938228 PMC10632506

[ref125] Wang J. X. Chang S. Y. Jin Z. Y. Li D. Zhu J. Luo Z. B. . (2025). *Lactobacillus reuteri*-enriched eicosatrienoic acid regulates glucose homeostasis by promoting GLP-1 secretion to protect intestinal barrier integrity. J. Agric. Food Chem. 73, 393–408. doi: 10.1021/acs.jafc.4c03818, 39680859

[ref126] Wang K. Zhao H. Zhao X. Zhang X. Zhang W. Cheng Y. . (2024). Photobiomodulation for diabetes and its complications: a review of general presentation, mechanisms and efficacy. Ann. Med. 56:2433684. doi: 10.1080/07853890.2024.2433684, 39607829 PMC11610354

[ref127] Wang Q. Lin H. Shen C. Zhang M. Wang X. Yuan M. . (2023c). Gut microbiota regulates postprandial GLP-1 response via ileal bile acid-TGR5 signaling. Gut Microbes 15:2274124. doi: 10.1080/19490976.2023.2274124, 37942583 PMC10730136

[ref128] Wu X. Park S. (2022). Fecal Bacterial Community and Metagenome Function in Asians with Type 2 Diabetes, According to Enterotypes. Biomedicine 10:2998. doi: 10.3390/biomedicines10112998, 36428566 PMC9687834

[ref129] Xie C. Qi C. Zhang J. Wang W. Meng X. Aikepaer A. . (2025). When short-chain fatty acids meet type 2 diabetes mellitus: Revealing mechanisms, envisioning therapies. Biochem. Pharmacol. 233:116791. doi: 10.1016/j.bcp.2025.116791, 39894305

[ref130] Xie X. Zhang M. Luo H. (2024). Regulation of metabolism by circadian rhythms: Support from time-restricted eating, intestinal microbiota & omics analysis. Life Sci. 351:122814. doi: 10.1016/j.lfs.2024.122814, 38857654

[ref131] Xu M. Dang S. Xin N. (2022). Correlations among intestinal flora levels with IL-6, TNF-α and HOMA-IR levels in patients with type 2 diabetes mellitus. Med. J. Chin. People's Health 34, 8–14. doi: 10.3969/j.issn.1672-0369.2022.10.003

[ref132] Xu P. Morishige J. I. Jing Z. Nagata N. Shi Y. Iba T. . (2024). Exenatide administration time-dependently affects the hepatic circadian clock through glucagon-like peptide-1 receptors in the central nervous system. Biochem. Pharmacol. 230:116567. doi: 10.1016/j.bcp.2024.116567, 39369911

[ref133] Xu W. Bo J. Jia L. Zhu K. Luo Q. (2025). Melatonin Confers Protection Against Multidrug-Resistant Bacterial Infections in Aged Mice Via Microbiota-Derived Butyrate. J. Pineal Res. 77:e70087. doi: 10.1111/jpi.70087, 41063483

[ref134] Yan S. Qin Q. Chen J. Yang Y. Yan H. Li T. . (2022). Analysis on differences in gut microbiota of type 2 diabetes patients with different levels of glycemic control. J. Zhengzhou Univ. 57, 238–243. doi: 10.13705/j.issn.1671-6825.2021.04.082

[ref135] Yang D. F. Huang W. C. Wu C. W. Huang C. Y. Yang Y. S. H. Tung Y. T. (2023). Acute sleep deprivation exacerbates systemic inflammation and psychiatry disorders through gut microbiota dysbiosis and disruption of circadian rhythms. Microbiol. Res. 268:127292. doi: 10.1016/j.micres.2022.127292, 36608535

[ref136] Yang Y. Zhang J. (2020). Bile acid metabolism and circadian rhythms. Am. J. Physiol. Gastrointest. Liver Physiol. 319, G549–g563. doi: 10.1152/ajpgi.00152.2020, 32902316

[ref137] Zambrano C. Garitaonaindia M. T. Salmerón D. Pérez-Sanz F. Tchio C. Picinato M. C. . (2024). Melatonin decreases human adipose tissue insulin sensitivity. J. Pineal Res. 76:e12965. doi: 10.1111/jpi.12965, 38860494

[ref138] Zarezadeh M. Musazadeh V. Faghfouri A. H. Sarmadi B. Jamilian P. Jamilian P. . (2022). Probiotic therapy, a novel and efficient adjuvant approach to improve glycemic status: An umbrella meta-analysis. Pharmacol. Res. 183:106397. doi: 10.1016/j.phrs.2022.106397 35981707

[ref139] Zhai T. Zou X. Zhang Z. Wang Y. Shi L. Ren W. . (2025). Circadian rhythms of gut microbiota and plaque vulnerability: mechanisms and chrono-microbiota modulation interventions. Gut Microbes 17:2532703. doi: 10.1080/19490976.2025.2532703, 40650475 PMC12258220

[ref140] Zhang C. Jiang J. Wang C. Li S. Yu L. Tian F. . (2022). Meta-analysis of randomized controlled trials of the effects of probiotics on type 2 diabetes in adults. Clin. Nutr. 41, 365–373. doi: 10.1016/j.clnu.2021.11.037, 34999331

[ref141] Zhang E. E. Liu Y. Dentin R. Pongsawakul P. Y. Liu A. C. Hirota T. . (2010). Cryptochrome mediates circadian regulation of cAMP signaling and hepatic gluconeogenesis. Nat. Med. 16, 1152–1156. doi: 10.1038/nm.2214, 20852621 PMC2952072

[ref142] Zhang L. Wei J. Liu X. Li D. Pang X. Chen F. . (2025). Gut microbiota-astrocyte axis: new insights into age-related cognitive decline. Neural Regen. Res. 20, 990–1008. doi: 10.4103/nrr.Nrr-d-23-01776, 38989933 PMC11438350

[ref143] Zhang Y. Li Y. Barber A. F. Noya S. B. Williams J. A. Li F. . (2023). The microbiome stabilizes circadian rhythms in the gut. Proc. Natl. Acad. Sci. USA 120:e2217532120. doi: 10.1073/pnas.2217532120, 36689661 PMC9945975

[ref144] Zhen Y. Ge L. Xu Q. Hu L. Wei W. Huang J. . (2022). Normal Light-Dark and Short-Light Cycles Regulate Intestinal Inflammation, Circulating Short-chain Fatty Acids and Gut Microbiota in Period2 Gene Knockout Mice. Front. Immunol. 13:848248. doi: 10.3389/fimmu.2022.848248, 35371053 PMC8971677

[ref145] Zielinski M. R. Gibbons A. J. (2022). Neuroinflammation, sleep, and circadian rhythms. Front. Cell. Infect. Microbiol. 12:853096. doi: 10.3389/fcimb.2022.853096, 35392608 PMC8981587

